# Projections from Regions of the Cerebellar Nuclei Receiving Jaw Muscle Proprioceptive Signals to Trigeminal Motoneurons and Their Premotoneurons in the Rat Pons and Medulla

**DOI:** 10.1007/s12311-025-01862-7

**Published:** 2025-06-12

**Authors:** Fumihiko Sato, Yumi Tsutsumi, Ayaka Oka, Takahiro Furuta, Jaerin Sohn, Yuki Oi, Mai Amano, Akiko Morita, Katsuro Uchino, Takafumi Kato, Yong Chul Bae, Yoshihisa Tachibana, Barry J. Sessle, Atsushi Yoshida

**Affiliations:** 1 Department of Systematic Anatomy and Neurobiology, Osaka University Graduate School of Dentistry, Suita, Osaka 565-0871 Japan; 2 Department of Orthodontics and Dentofacial Orthopedics, Osaka University Graduate School of Dentistry, Suita, Osaka 565-0871 Japan; 3 Department of Acupuncture, Faculty of Health Care Sciences, Takarazuka University of Medical and Health Care, Takarazuka, Hyogo 666-0162 Japan; 4 Department of Physical Therapy, Faculty of Health Care Sciences, Takarazuka University of Medical and Health Care, Takarazuka, Hyogo 666-0162 Japan; 5 Department of Oral Health Sciences, Faculty of Health Care Sciences, Takarazuka University of Medical and Health Care, Takarazuka, Hyogo 666-0162 Japan; 6 Department of Oral Physiology, Osaka University Graduate School of Dentistry, Suita, Osaka 565-0871 Japan; 7 Department of Anatomy and Neurobiology, School of Dentistry, Kyungpook National University, Daegu, 700-412 Korea; 8 Division of Physiology and Cell Biology, Kobe University Graduate School of Medicine, Kobe, Hyogo 650-0017 Japan; 9 Faculty of Dentistry and Department of Physiology, Temerty Faculty of Medicine, University of Toronto, Toronto, ON M5G 1G6 Canada

**Keywords:** Cerebellofugal, Interposed cerebellar nucleus, Medial cerebellar nucleus, Muscle spindle, Mastication

## Abstract

The cerebellum plays a crucial role in sensorimotor control through cerebellofugal projections from the cerebellar nuclei. However, little is known about the cerebellofugal projection features involved in jaw sensorimotor control, although the dorsolateral parts of the interposed cerebellar nucleus (IntDL) and medial cerebellar nucleus (MedDL) do receive proprioceptive signals bilaterally from rat jaw-closing muscle spindles (JCMSs). This study aimed to detail the cerebellofugal projection features involved in jaw sensorimotor control. Anterograde tracer was injected into regions of the rat IntDL and MedDL receiving JCMS proprioceptive inputs (i.e., jcms-IntDL and jcms-MedDL). Axon terminals arising from the jcms-IntDL were labeled bilaterally with an ipsilateral predominance in several pontomedullary regions, although very few terminals were labeled in the dorsolateral and ventromedial divisions (5dl and 5vm) of the trigeminal motor nucleus. In contrast, terminals from the jcms-MedDL were labeled bilaterally with a contralateral predominance in several pontomedullary regions and a few terminals were labeled in the contralateral 5dl and 5vm. Thus, the projections from the jcms-IntDL and jcms-MedDL were well segregated. Subsequent retrograde tracer injections into the pontomedullary regions demonstrated that amongst the entire cerebellar nuclei the nucleofugal projections principally arose from the IntDL and MedDL. Additionally, many premotoneurons for the 5dl or 5vm were widely labeled in the pontomedullary regions where many axons from the jcms-IntDL or jcms-MedDL terminated. The various connections involving the jcms-IntDL and jcms-MedDL may play a crucial role in jaw sensorimotor control, mainly through indirect cerebellofugal pathways to the 5dl and 5vm via their premotoneurons.

## Introduction

The cerebellum is crucial for sensorimotor control [1–9]. Since cerebellofugal projections arise exclusively from neurons in the cerebellar nuclei, cerebellar sensorimotor modulation may involve these projections from the cerebellar nuclei to motoneurons in the lower brainstem or spinal cord. However, the cerebellofugal projection features involved in cerebellar sensorimotor control are still largely unclear. Nonetheless, it is well known that cerebellar sensorimotor control involves the use of sensory inputs such as those from muscle spindles. Muscle proprioceptive signals provide information about muscle length and state of contraction, and regulate the activity of motoneurons in the lower brainstem or spinal cord through mono- or poly-synaptic reflex arcs (e.g., [10–13]). Thus, it is highly likely that the neurons of the cerebellar nuclei giving rise to the cerebellofugal projections receive muscle proprioceptive afferent inputs that are utilized for cerebellar sensorimotor control. Previous studies [14–17] have indeed shown that the interposed cerebellar nucleus (Int) receives muscle proprioceptive afferent inputs from forelimb, hindlimb and masseter muscles in cats, but these previous studies did not demonstrate the exact location of their recording sites in the Int and the sites receiving cerebellofugal projections from the recording sites in the Int.

Likewise in the craniofacial region, the cerebellofugal projection patterns involved in jaw sensorimotor control are largely unclear. Muscle spindles are abundant in the jaw-closing (JC) muscles, but are few or absent in the jaw-opening (JO) muscles and other craniofacial muscles such as facial, laryngopharyngeal, and lingual muscles of subprimates [10, 11, 18–20]. The proprioceptive signals arising from the JC muscle spindles (JCMSs) are well known to be conveyed by the trigeminal mesencephalic nucleus (Me5) neurons (primary afferent neurons innervating muscle spindles in JC muscles) to the dorsolateral division (5dl) of the trigeminal motor nucleus (Mo5, which contains JC motoneurons innervating the JC muscles), as well as to the supratrigeminal nucleus (Su5, which is located dorsolateral to the 5dl); the ventromedial division (5vm) of the Mo5 contains JO motoneurons innervating the JO muscles [10–12, 21, 22]. The JC motoneurons in the 5dl do not have axon branches, which convey JCMS proprioceptive signals to the brainstem areas outside the 5dl [22–25]. Therefore, to identify the cerebellar nuclei receiving JCMS proprioceptive afferent inputs in the rat, we recently used both anterograde and retrograde labeling techniques to examine direct projections from the Su5 to the cerebellar nuclei, and showed that the Su5 directly projects bilaterally to the dorsolateral hump (IntDL) of the Int and the dorsolateral protuberance (MedDL) of the medial cerebellar nucleus (Med) in our previous study of Tsutsumi et al. [26]; the IntDL and MedDL are cytoarchitectonically prominent, respectively, in the Int and Med of rodents, although not in higher animal species [27–30]. In the study of Tsutsumi et al. [26], we have electrophysiologically demonstrated that some regions of the rat IntDL and MedDL contain neurons receiving proprioceptive afferent inputs from the JCMS; we have referred to these as jcms-IntDL and jcms-MedDL, respectively. However, the cerebellofugal pathways from the rat jcms-IntDL and jcms-MedDL to the Mo5 as well as their connections with other pontomedullary regions remain unclear. Therefore, the aim of this study was to detail the cerebellofugal projection features involved in jaw sensorimotor control by demonstrating the connections of these two cerebellar nuclei with the Mo5 and other pontomedullary regions in the rat. For this purpose, we combined electrophysiological recordings of JCMS evoked responses in the IntDL and MedDL with anterograde and retrograde labeling techniques applied to these nuclei and pontomedullary regions.

## Materials and Methods

### Animals

Experiments were conducted on 49 young adult, male Wistar rats weighing 250–330 g. While sex differences in brain organization, such as the size of specific brain regions and the release of neurotransmitters and hormones, have been reported [31–33], we used male rats in this study to maintain consistency and to facilitate comparison with previous relevant studies [26, 34, 35] which used only young adult male rats. All experimental procedures for the care and use of laboratory animals were approved by the animal ethics committees of the Osaka University Graduate School of Dentistry. Efforts were made to minimize animal suffering and the number of animals used.

### Surgery

All animals were continuously anesthetized by inhalation of 3% isoflurane delivered via a nose cone such that neither spontaneous eye movements nor corneal reflexes were apparent. A local injection of lidocaine hydrochloride was administered before making a skin incision, if necessary. The rectal temperature was maintained at 37–38 ºC with a heating pad, and electrocardiography was performed continuously.

Three experiments were conducted in this study. In the first and second experiments, 17 and 32 rats were used, respectively (Tables 1, 2). In all of the 17 rats used in the first experiment (Table 1), the masseter nerve, which innervates the masseter muscle (one of the JC muscles) on the right side, was exposed to allow for electrical stimulation of the trigeminal mesencephalic nucleus primary afferents which innervate the muscle spindles in the masseter muscle as in previous studies [21, 22, 26, 36–42]. This approach has been shown to effectively activate the afferents and evoke orthodromically activated responses recorded from the cerebellar nucleus neurons [26]. In 17 of the 32 rats used in the second experiment (Table 2) for injections of a retrograde tracer into the 5dl of the Mo5 (which contains JC motoneurons) or its adjacent regions as described below, the right masseter nerve was exposed to allow for effective electrical stimulation of the motor fibers in the masseter nerve in order to and evoke antidromically activated responses recorded from the masseter motoneurons so as to locate the 5dl, as previously described [35, 43, 44]. In another nine rats of the 32 rats in the second experiment (Table 2), for injections of a retrograde tracer into the 5vm of the Mo5 (which contains JO motoneurons) or its adjacent region as described below, the mylohyoid nerve, which innervates the anterior belly of the digastric muscle (one of the JO muscles) on the right side, was exposed to allow for effective electrical stimulation of the motor fibers in the mylohyoid nerve and evoke antidromically activated responses recorded from JO motoneurons in order to locate the 5vm, as previously described [35, 43, 44]. For electrical stimulation, the masseter and mylohyoid nerves were dissected free from the surrounding muscles and other tissues, and silver bipolar stimulation electrodes were hooked around each of them. In addition, the remaining six rats of the 32 rats in the second experiment were used without stimulation of the masseter and mylohyoid nerves (Table 2). Subsequently, in all of the rats used in the first and second experiments, the animal’s head was placed in a stereotaxic apparatus. A glass micropipette was inserted into the brain after small parts of the cranial bone and the dura overlying target structures on the right side were removed. The rat brain atlases published by Paxinos and Watson [45, 46] were primarily used to approximate the stereotaxic coordinates.

**Table 1:** Summary of the First Experiment

Total No. of animals	Target structurer	No. of animals with electrophysiological responses (= No. of animals used for BDA injections)	Center of BDA deposit (= Analysis of recordings)	No. of animals	Extent of BDA deposit	No. of animals	No. of animals used for the analysis of BDA labeled axons
17	IntDL	8	Inside IntDL	5	Within IntDL	3	3
					Beyond IntDL	2	
			Outside IntDL	3			
	MedDL	9	Inside MedDL	4	Within MedDL	2	2
					Beyond MedDL	2	
			Outside MedDL	5			

All 17 rats received electrical stimulation of the masseter nerve

**Table 2:** Summary of the Second and Third Experiments

No. of animals used in the second experiment	Target structure of CTb injection	Site of electrical stimulation	Center of CTb deposit	No. of animals	Extent of CTb deposit	No. of animals	No. of animals used in the third experiment
32	5dl	Masseter nerve	Inside 5dl	5	Within 5dl	5	5
					Beyond 5dl	0	
			Outside 5dl	0			
	5vm	Mylohyoid nerve	Inside 5vm	5	Within 5vm	5	5
					Beyond 5vm	0	
			Outside 5vm	0			
	Su5	Masseter nerve	Inside Su5	4	Within Su5	3	
					Beyond Su5	1	
			Outside Su5	0			
	I5	Masseter nerve	Inside I5	4	Within I5	3	
					Beyond I5	1	
			Outside I5	0			
	Rm5vm	Mylohyoid nerve	Inside Rm5vm	4	Within Rm5vm	3	
					Beyond Rm5vm	1	
			Outside Rm5vm	0			
	LRF medial to the 5Or	Masseter nerve	Inside LRF medial to the 5Or	4	Within LRF medial to the 5Or	3	
					Beyond LRF medial to the 5Or	1	
			Outside LRF medial to the 5Or	0			
	LRF in the caudal pons		Inside LRF in the caudal pons	3	Within LRF in the caudal pons	3	
					Beyond LRF in the caudal pons	0	
			Outside LRF in the caudal pons	0			
	Dorsolateral GRF in the rostral medulla		Inside the dorsolateral GRF in the rostral medulla	3	Within the dorsolateral GRF in the rostral medulla	3	
					Beyond the dorsolateral GRF in the rostral medulla	0	
			Outside the dorsolateral GRF in the rostral medulla	0			

In the first experiment, we electrophysiologically recorded orthodromically activated responses evoked by the masseter nerve stimulation to determine the JCMS projection sites in the cerebellar nuclei for which we also subsequently identified with anterograde tract tracing their cerebellofugal projection features. We vertically inserted a glass electrode filled with an anterograde tracer biotinylated dextran amine (BDA; 4%; 10,000 MW; Molecular Probes, Eugene, OR, USA) dissolved in 0.01 M phosphate buffer (PB; pH 7.4) into the right cerebellum to target the jcms-IntDL or jcms-MedDL, as previously described [26]. The location of each BDA-injection site was determined by electrophysiological recordings of short-latency, large field potentials evoked by electrical stimulation of the masseter nerve (200-μs duration single pulse, interval of 1 s), and/or multi-unit responses to passive, sustained jaw-opening, which causes proprioceptive inputs into the CNS from the JC muscles, as previously described [26]. At the recording sites, we electrophoretically made a single injection of BDA into the jcms-IntDL in eight rats and into the jcms-MedDL in nine rats (Table 1) by delivering 2.0-μA positive pulses (300-ms duration single pulse, interval of 500 ms) for 5–10 min.

In the second experiment, we used electrophysiological recordings and subsequent retrograde tract-tracing to examine the distribution of the cerebellar nuclear neurons projecting to those pontomedullary areas where anterogradely labeled axon terminals were mainly found after BDA injections into the jcms-IntDL and jcms-MedDL in the first experiment. We used a glass micropipette filled with a retrograde tracer cholera toxin B subunit (CTb; 1%; List Biological Laboratories, Campbell, CA, USA) dissolved in 0.1 M PB as previously described [39, 41]. The glass micropipette was inserted into the occipital cortex obliquely at an 18º rostral-to-caudal inclination to target the 5dl, 5vm, Su5, intertrigeminal region (I5) (reticular region between the trigeminal principal nucleus [Pr5] and the Mo5), the reticular region medial to the 5vm (Rm5vm), and the lateral reticular formation (LRF) medial to the rostro-dorsomedial part (5Or) of the trigeminal oral subnucleus (5O) in the pons. The exact location of the 5dl and 5vm was determined by using the micropipette to record large short-latency antidromic field potentials evoked by electrical stimulation (200-μs duration single pulse, interval of 1 s) of the masseter nerve and the mylohyoid nerve, respectively, as in our previous studies [35, 43]. For injections into the Su5, orthodromic, disynaptic responses evoked by electrical stimulation of the masseter nerve were recorded with the micropipette as previously described [36, 37]. For targeting the I5 and LRF medial to the 5Or, the electrode was slightly moved lateral and caudoventral to the 5dl, respectively. For targeting the Rm5vm, the micropipette was moved ventromedial to the 5vm. For targeting the LRF in the caudal pons and the dorsolateral gigantocellular reticular formation (GRF) in the rostral medulla, we inserted the micropipette obliquely at a 45º caudo-to-rostral inclination into the caudal pons or rostral medulla through its dorsal surface, after gently lifting the posterior cerebellum and slightly pushing it rostrally. Finally, a single injection of CTb was carried out electrophoretically by delivering 2.0-μA positive pulses (300-ms duration single pulse, interval of 500 ms) for 5–20 min into the 5dl in five rats, 5vm in five rats, Su5 in four rats, I5 in four rats, Rm5vm in four rats, LRF medial to the 5Or in four rats, LRF in the caudal pons in three rats, and dorsolateral GRF in the rostral medulla in three rats (Table 2).

In the third experiment, we neither recorded any electrophysiological responses nor made any tracer injections. Instead, we analyzed the distribution of retrogradely CTb-labeled premotoneurons projecting to the 5dl or 5vm in the pons and medulla by microscopically observing the specimens already obtained after CTb injections into the 5dl (in five rats) and 5vm (in another five rats) in the second experiment described above (Table 2).

After the tracer injections in the first and second experiments, the glass micropipette was carefully withdrawn, and the stimulation electrodes were detached from the masseter or mylohyoid nerve. All wounds were then sutured. Next, analgesic (flurbiprofen axetil, 3.3 mg/kg) and an antibiotic (cefotiam hydrochloride, 66 mg/kg) were administered intraperitoneally, and the animals were allowed to recover from anesthesia in their cages as previously described [40, 41]. During postinjection survival, the rats were monitored on a daily basis to assess their general behaviors, body weight, and any postoperative complications such as bleeding or inflammation.

### Histology

After a post-injection survival of 5–7 days for rats injected with BDA in the first experiment or 4–5 days for rats injected with CTb in the second experiments, the rats were deeply anesthetized with sodium pentobarbital (100 mg/kg, i.p.). As in our previous studies [26, 42], rats were perfused with 100 ml of saline followed by 300 ml of a fixative containing 4% paraformaldehyde in 0.1 M PB through the ascending aorta. Then, the cerebellum, pons and medulla were removed and placed in 25% sucrose in 0.1 M PB at 4 °C for a few days, until these had sunk. They were embedded in 10% gelatin dissolved in 0.1 M PB, and were then cut coronally at 60 μm thick on a freezing microtome. The serial sections were alternately divided into three sets. For the detection of BDA in the first experiment, all sets of alternate serial sections were washed in 0.02 M PBS (pH 7.4) and preincubated in 0.02 M PBS containing 0.01% H_2_O_2_ and 0.75% Triton X-100, as previously described [41, 47]. In control cases where the BDA was not applied, no labeling was detected. For the visualization of CTb in the second experiment, all sets of sections were preincubated in 0.02 M PBS containing 3% normal goat serum, 0.2% Triton X-100, and polyclonal rabbit anti-CTb primary antibody (GeneTex, Alton Pkwy Irvine, CA, USA) diluted to 1:20,000. The sections were then incubated in 0.02 M PBS containing biotinylated goat anti-rabbit immunoglobulin G diluted to 1:400. In control cases where the CTb was not applied or the primary antibody was omitted, no labeling was detected. Subsequently, all sections from all rats with BDA or CTb injections were incubated in 0.02 M PBS containing avidin–biotin–peroxidase complex diluted at 1:100, and were then placed in a diaminobenzidine solution (0.1 M PB [pH 7.4] containing 0.04% diaminobenzidine, 0.006% H_2_O_2_ and 0.08% nickel ammonium sulfate). The sections were then mounted on gelatin-coated slides and dried, and one set of sections was counterstained with Neutral Red. Finally, all sections were dehydrated in graded alcohols, cleared in xylene, and coverslipped.

### Data Analysis

In the first and second experiments, the recorded field potentials were stored on a computer, and offline analysis was performed with computer assistance (PowerLab 8/30, ADInstruments, Sydney, Australia) as described in our previous studies [38–40]. Responses to six to nine successive peripheral stimuli were averaged at each recording site. In all of three experiments, we used a camera lucida attached to a light microscope in order to visualize and draw the brain structures, BDA injection sites, anterogradely BDA-labeled axonal fibers and terminals, CTb injection sites, or retrogradely CTb-labeled neuronal cell bodies.

## Results

### Anterogradely Labeled Axon Fibers and Terminals from the jcms-IntDL and jcms-MedDL

**Fig. 1:**
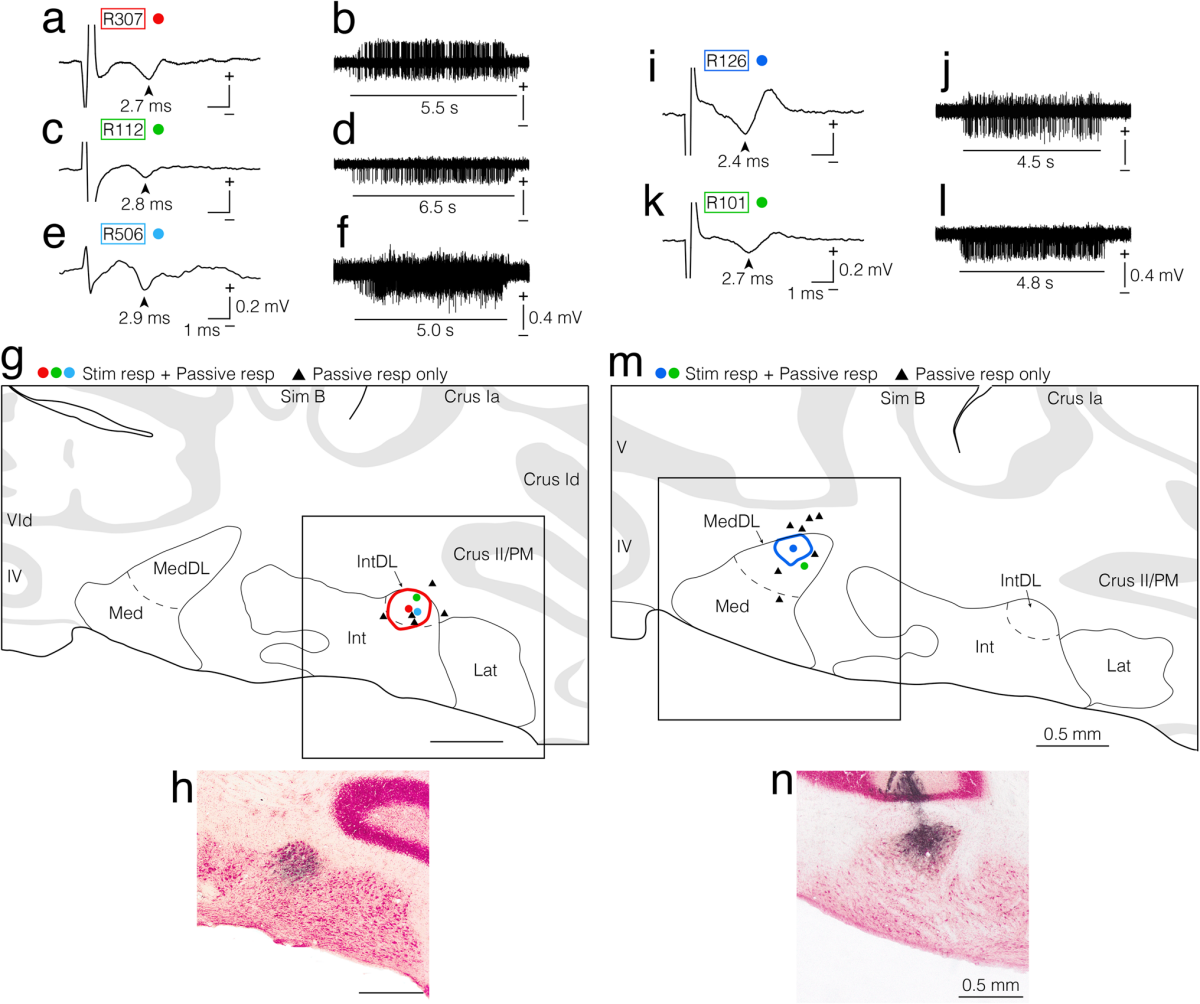
Electrophysiologically evoked responses recorded in the dorsolateral hump of the interposed cerebellar nucleus (IntDL) (**a–f**) and the dorsolateral protuberance of the medial cerebellar nucleus (MedDL) (**i–l**) of the cerebellar nuclei on the right side ipsilateral to the electrically stimulated masseter nerve and injection sites of anterograde tracer biotinylated dextran amine (BDA) made at the recording sites (**g**, **h**, **m**, **n**). **a**, **c**, **e**, **i**, **k** Field potentials evoked by electrical stimulation of the ipsilateral masseter nerve. Arrowheads indicate peak responses with approximate latencies of 2.7 ms (**a**), 2.8 ms (**c**), 2.9 ms (**e**), 2.4 ms (**i**) and 2.7 ms (**k**). **b**, **d**, **f, j, l** Extracellular multi-unit discharges recorded during a sustained jaw-opening (stretching the muscle spindles of the jaw-closing (JC) muscles [JCMSs]). Horizontal lines in panels **b**, **d**, **f, j, l** indicate durations of jaw-opening (approximately 5.5 s, 6.5 s, 5.0 s, 4.5 s and 4.8 s, respectively). Note that each recording site corresponds to the center of BDA-deposit that was subsequently injected. **g**, **m** Drawings of coronal sections including the cerebellar nuclei. In rat R307, both responses **a** and **b** were recorded at the site indicated by a red dot in the IntDL in panel **g**. The extent of the BDA-deposit in this rat is indicated by a red circle in panel **g** and is shown in photomicrograph **h**. The boxed area in panel **g** corresponds to the area shown in photomicrograph **h**. In rat R112, both responses **c** and** d** were recorded at the site indicated by a green dot in the IntDL in panel **g**. In rat R506, both responses **e** and** f** were recorded at the site indicated by a sky-blue dot in the IntDL in panel **g**. In rat R126, both responses **i** and **j** were recorded at the site indicated by a blue dot in the MedDL in panel **m**. The extent of the BDA-deposit in this rat is indicated by a blue circle in panel **m** and is shown in photomicrograph **n**. The boxed area in panel **m** corresponds to the area shown in photomicrograph **n**. In rat R101, both responses **k** and** l** were recorded at the site indicated by a green dot in the MedDL in panel **m**. In panels **g** and **m**, recording sites (the centers of BDA deposits) where both types of responses described above were recorded are labeled as “Stim resp + Passive resp” and indicated by colored dots. Sites where only the extracellular multi-unit discharges during sustained jaw-opening were recorded are labeled as “Passive resp only” and indicated by filled triangles. For abbreviations, see the abbreviations list. Scale bars = 1 ms and 0.2 mV in (**a**), (**c**) and (**i**) as in (**e**) and (**k**); 0.4 mV in (**b**), (**d**) and (**j**) as in (**f**) and (**l**); 0.5 mm in (**g**) and (**h**) as in (**m**) and (**n**)

**Fig. 2:**
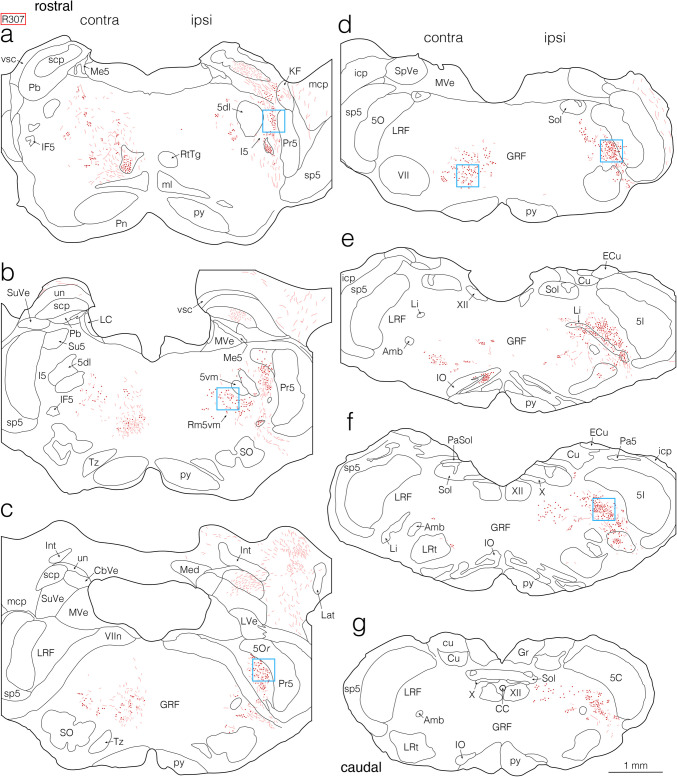
Semi-schematic drawings showing the distribution of anterogradely BDA-labeled axon fibers and terminals in the pons and medulla after a BDA injection into the jcms-IntDL in rat R307. **a–g** Drawings of seven coronal sections arranged rostrocaudally from (**a**) to (**g**). Left and right sides of each panel correspond respectively to the sides contralateral (contra) and ipsilateral (ipsi) to the BDA injection site in the jcms-IntDL. As shown in Fig. 1g, h, the injection site was located at the caudal level of the pons (slightly rostral to the level shown in panel [**d**]). **b**, **c** Axon fibers and terminals labeled in the medullary body of the cerebellar white matter, which includes the cerebellar nuclei, are drawn, but those labeled in the cerebellar cortex and the white plate of the cerebellar white matter are not drawn. Boxed areas in (**a**), (**b**), (**c**), (**d**, right box), (**d**, left box), and (**f**), which included axon terminals labeled in the ipsilateral intertrigeminal region (I5), ipsilateral reticular region (Rm5vm) medial to the ventromedial division (5vm) of the trigeminal motor nucleus (Mo5), ipsilateral lateral reticular formation (LRF) medial to the rostro-dorsomedial part (5Or) of the trigeminal oral subnucleus (5O), ipsilateral LRF in the caudal pons, contralateral gigantocellular reticular formation (GRF) in the caudal pons, and ipsilateral LRF in the rostral medulla, respectively, correspond to the areas taken in photomicrographs (**a–f**) in Fig. 4, respectively. For abbreviations, see the abbreviations list. Scale bar = 1 mm in (**g**) (also applies to (**a**)-(**f**))

**Fig. 3:**
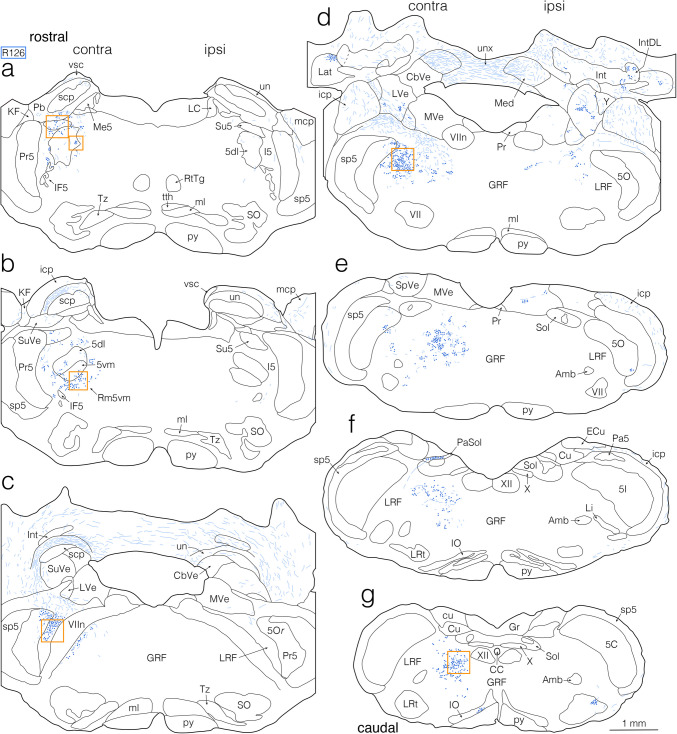
Semi-schematic drawings showing the distribution of anterogradely BDA-labeled axon fibers and terminals in the pons and medulla after a BDA injection into the jcms-MedDL in rat R126. **a–g** Drawings of seven coronal sections arranged rostrocaudally from (**a**) to (**g**). Left and right sides of each panel correspond respectively to the sides contralateral (contra) and ipsilateral (ipsi) to the BDA injection site in the jcms-MedDL. The injection site is shown in Fig. 1m, n, and was located at the rostrocaudally middle level of the pons (between two levels shown in panels [**d**] and [**e**]). **c**, **d** Axon fibers and terminals labeled in the medullary body of the cerebellar white matter, which includes the cerebellar nuclei, are drawn, but those labeled in the cerebellar cortex and the white plate of the cerebellar white matter are not drawn. Boxed areas in (**a**, upper left box), (**a**, lower right box), (**b**), (**c**), (**d**), and (**g**), which included axon terminals labeled in the contralateral supratrigeminal nucleus (Su5), contralateral dorsolateral division of the Mo5 (5dl), contralateral 5vm and Rm5vm, contralateral LRF medial to the 5Or, contralateral LRF in the caudal pons, and contralateral, dorsolateral GRF in the medulla, respectively, correspond to the areas taken in photomicrographs (**g–l**) in Fig. 4, respectively. For abbreviations, see the abbreviations list. Scale bar = 1 mm in (**g**) (also applies to (**a**)-(**f**))

In the first experiment, we used electrophysiological techniques to locate the jcms-IntDL and jcms-MedDL in the IntDL and MedDL, respectively. Electrophysiological identification of the jcms-IntDL and jcms-MedDL was achieved by determining whether the recording sites responded to electrical stimulation of the ipsilateral masseter nerve containing the primary afferents innervating the JCMSs (e.g., Fig. 1a, c, e, i, k) and/or to passive, sustained jaw-opening (e.g., Fig. 1b, d, f, j, l), as previously described [26]. We subsequently made BDA injections at the recording sites to observe the distribution of axon terminals originating from these regions of the IntDL and MedDL receiving JCMS afferent inputs, respectively (e.g., Figs. 2, 3); the BDA injections were made in eight IntDL-targeted rats and nine MedDL-targeted rats (Table 1). In all 17 rats, multi-unit responses to passive, sustained jaw-opening could be recorded (e.g., Fig. 1b, d, f, j, l); in addition, short-latency, large field potential responses evoked by electrical stimulation of the masseter nerve were also observed in three rats (R307, R112 and R506) of the IntDL group (Fig. 1a, c, e), and two rats (R126 and R101) of the MedDL group (Fig. 1i, k). The peak potential latencies in response to electrical stimulation were 2.7 ms (R307), 2.8 ms (R112), and 2.9 ms (R506) for the IntDL, and 2.4 ms (R126) and 2.7 ms (R101) for the MedDL. These latencies were consistent with those reported in previous studies targeting the jcms-IntDL and jcms-MedDL [26]. The exact recording sites were histologically confirmed based on the location of the centers of the injected BDA deposits. Table 1 outlines the centers and extents of the BDA deposits injected into the IntDL or MedDL sites from which the electrophysiologically evoked responses could be recorded in all 17 rats. Figure 1 shows that the recording sites in the three rats (R307, R112, and R506) of the IntDL group were clearly within the IntDL, and the extents of the injected BDA deposits were histologically confirmed to be confined to the IntDL in these three rats (Fig. 1g, h). The recording sites in the two rats (R126 and R101) of the MedDL group were clearly within the MedDL (Fig. 1m, n), and the extents of the injected BDA deposits were histologically confirmed to be confined to the MedDL in these two rats (Fig. 1m, n). Among the remaining five rats used for targeting the IntDL, two rats had injection centers within the IntDL, although the BDA deposits extended beyond the IntDL, but in the other three rats, the injection centers were located outside the IntDL (Fig. 1g). Similarly, for the seven rats used for targeting the MedDL, two rats had injection centers within the MedDL with some spread beyond the MedDL, whereas in the other five rats, the centers of BDA deposits were located outside the MedDL (Fig. 1m).

#### Axons from the jcms-IntDL

**Fig. 4:**
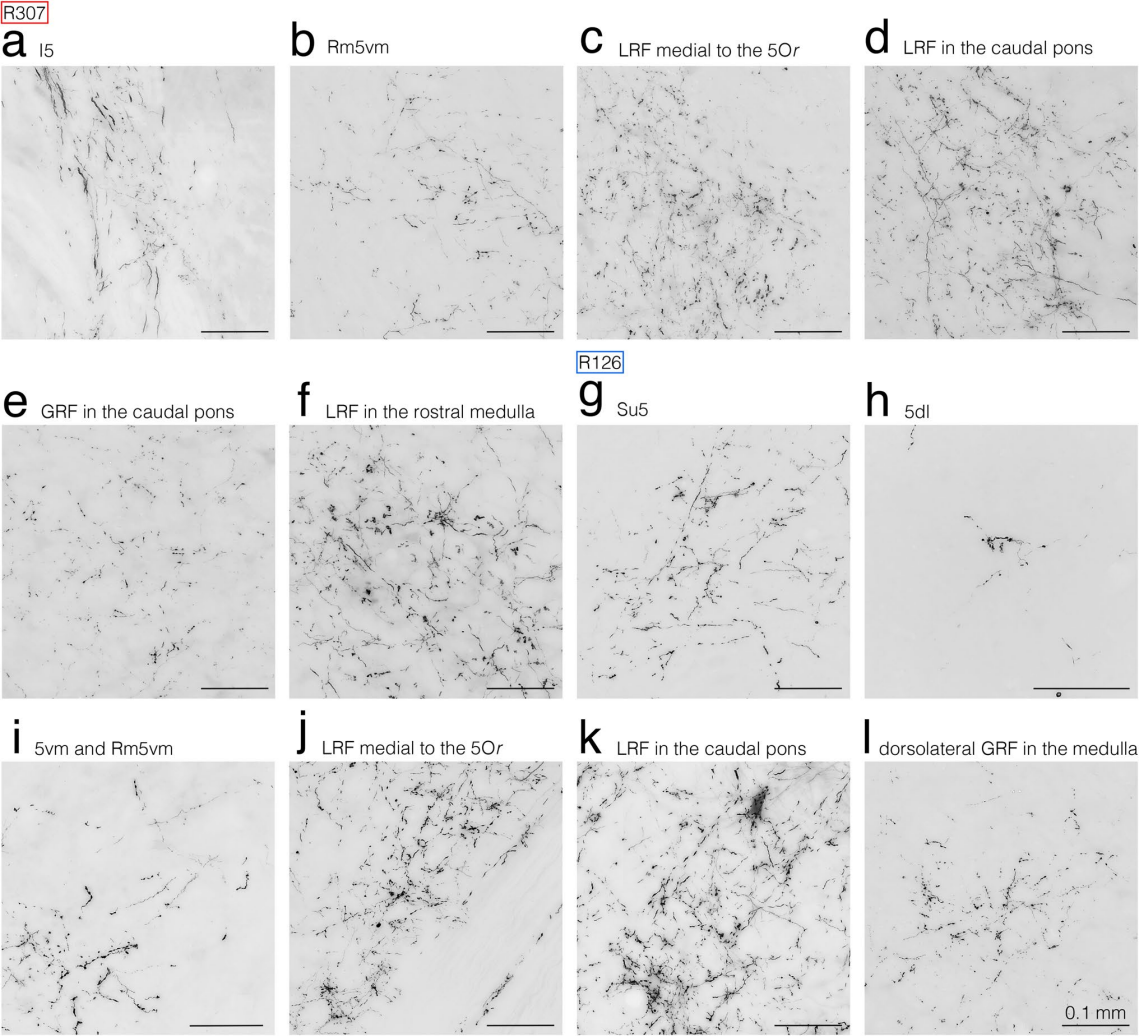
Photomicrographs showing axon fibers and terminals labeled in the ipsilateral I5 (**a**), ipsilateral Rm5vm (**b**), ipsilateral LRF medial to the 5Or (**c**), ipsilateral LRF in the caudal pons (**d**), contralateral GRF in the caudal pons (**e**), and ipsilateral LRF in the rostral medulla (**f**) in rat R307 receiving BDA injections into the jcms-IntDL, and those in the contralateral Su5 (**g**), contralateral 5dl (**h**), contralateral 5vm and Rm5vm (**i**), contralateral LRF medial to the 5Or (**j**), contralateral LRF in the caudal pons (**k**), and contralateral, dorsolateral GRF in the medulla (**l**) in rat R126 receiving BDA injections into the jcms-MedDL. Areas taken in photomicrographs (**a–l**) correspond to boxed areas in Fig. 2a, b, c, d (right box), d (left box), and f and Fig. 3a (upper left box), a (lower right box), b, c, d and g, respectively. For abbreviations, see the abbreviations list. Scale bars = 0.1 mm in (**a**)-(**k**) as in (**l**)

The three rats that received restricted BDA injections confined to the IntDL (R307, R112, and R506) exhibited a similar distribution of anterogradely BDA-labeled axon fibers and terminals (e.g., Fig. 2). In a representative rat R307, the center of the BDA deposit was located in the rostrocaudally middle of the IntDL at the middle level of the pons on the right side (Fig. 1g, h), between the levels shown in Fig. 2c and Fig. 2d. On the right side (ipsilateral to the BDA injection site), many BDA-labeled axon fibers emerged from the injection site and extended ventrolaterally to enter the superior cerebellar peduncle (scp) slightly rostral to the injection site (Fig. 2c). They ascended in the lateral part of the scp (Fig. 2b, c). At the rostral pontine level, these labeled axon fibers left the scp, extended ventrolaterally, and passed the parabrachial nucleus (Pb) to enter the I5 between the Pr5 of the trigeminal spinal nucleus and the Mo5 (Figs. 2a, b, 4a). These labeled axon fibers gave off many axon terminals in the I5 (Figs. 2a, b, 4a), and fewer in the interfascicular trigeminal nucleus (IF5), caudal Su5 (Fig. 2a, b) and Rm5vm (Figs. 2b, 4b); rarely were axon terminals labeled in the 5dl, 5vm, and Pr5 (Fig. 2a, b). Many axon fibers labeled in the I5 extended caudoventrally and gave off many labeled axon terminals in the LRF (the juxtatrigeminal region) medial to the 5Or of the trigeminal spinal nucleus and to the caudoventral Pr5 (Figs. 2c, 4c), and in the ventrolateral LRF medial to the ventral 5O of the trigeminal spinal nucleus at the caudal pontine level (Figs. 2d, 4d). No axon terminals were labeled in the 5Or, and only a few terminals were evident in the ventromedial 5O (Fig. 2c, d). In addition, only a few labeled terminals were observed in the caudo-dorsolateral VII (Fig. 2d). At the medullary level, labeled axon fibers in the LRF traveled caudally and terminated in the ventrolateral LRF medial to the interpolar subnucleus (5I) (Figs. 2e, f, 4f) and caudal subnucleus (5C) of the trigeminal spinal nucleus but not within these two subnuclei themselves (Fig. 2g). Labeled terminals were also seen in the linear nucleus of the medulla (Li) (Fig. 2e) and the rostro-dorsolateral part of the lateral reticular nucleus (LRt) (Fig. 2e, f). However, terminals were rarely found in the ambiguus nucleus (Amb) (Fig. 2e-g) and none in the XII and inferior olive (IO) (Fig. 2f, g). In addition, many labeled axon fibers in the lateral part of the ipsilateral scp at the rostral pontine level described above (Fig. 2a, b) further ascended within the scp and crossed the midline through the decussation of the scp at the caudal mesencephalic level (not shown in Fig. 2).

On the left side (contralateral side to the BDA injection site), many labeled axon fibers in the scp, after crossing the midline through the decussation of the scp, ascended towards the midbrain (not shown in Fig. 2). In contrast, many other labeled axon fibers left the scp, turned caudoventrally, and traveled caudoventrally in the reticular formation including the tectospinal tract at the caudal mesencephalon level, and, then at the rostral pontine level (Fig. 2a, b). Many labeled terminals were widely distributed in the pontine reticular formation, especially in its ventromedial regions (Fig. 2a, b), and aggregated in the caudal reticulotegmental nucleus of the pons (RtTg) (Fig. 2a). Some terminals were also labeled in the Rm5vm (Fig. 2b), but were rarely evident in the 5dl, 5vm, Su5, and Pr5. At the caudal pontine level, labeled fibers descended caudo-ventromedially in the ventromedial GRF and terminated in this reticular region, but not in the 5Or, 5O, LRF medial to the 5Or and 5O, and VII (Figs. 2c, d, 4e). At the rostral medulla level, some labeled fibers descended in the ventromedial GRF and terminated there (Fig. 2e). Labeled axon terminals were also aggregated in the dorsomedial part of the rostralmost level of the IO (Fig. 2e). However, axon fibers and terminals were rarely labeled in the middle and caudal levels of the medulla which include the Li, LRt, Amb and XII (Fig. 2f, g).

In summary, axon fibers and terminals arising from the jcms-IntDL were bilaterally labeled in the pons and medulla and had an ipsilateral predominance (Fig. 2). Despite the bilateral projections, the labeled terminals from the bilateral jcms-IntDL did not overlap, since the contralateral terminals were more medially distributed than the ipsilateral terminals.

#### Axons from the jcms-MedDL

In the two rats that received restricted BDA injections confined to the MedDL (R126 and R101), the anterogradely BDA-labeled axon fibers and terminals exhibited similar distribution (e.g., Fig. 3). In a representative rat R126, the center of the BDA deposit was located in the rostrocaudally middle MedDL (Fig. 1m, n) at the caudal pontine level (between levels shown in Fig. 3d and Fig. 3e) on the right side. Many BDA-labeled axon fibers emerged from the injection site and extended mainly in two directions: rostro-ventromedial and rostro-ventrolateral. The rostro-ventromedially extending labeled fibers further traveled rostromedially to cross the midline through the decussation (unx) of the uncinate fasciculus (un) (Fig. 3d). They then extended rostrolaterally on the contralateral side. In contrast to the labeled axon terminals arising from the jcms-IntDL, those from the jcms-MedDL were contralaterally dominant. Therefore, we first describe the features of labeled terminals on the contralateral side and subsequently describe those on the ipsilateral side.

**Fig. 5:**
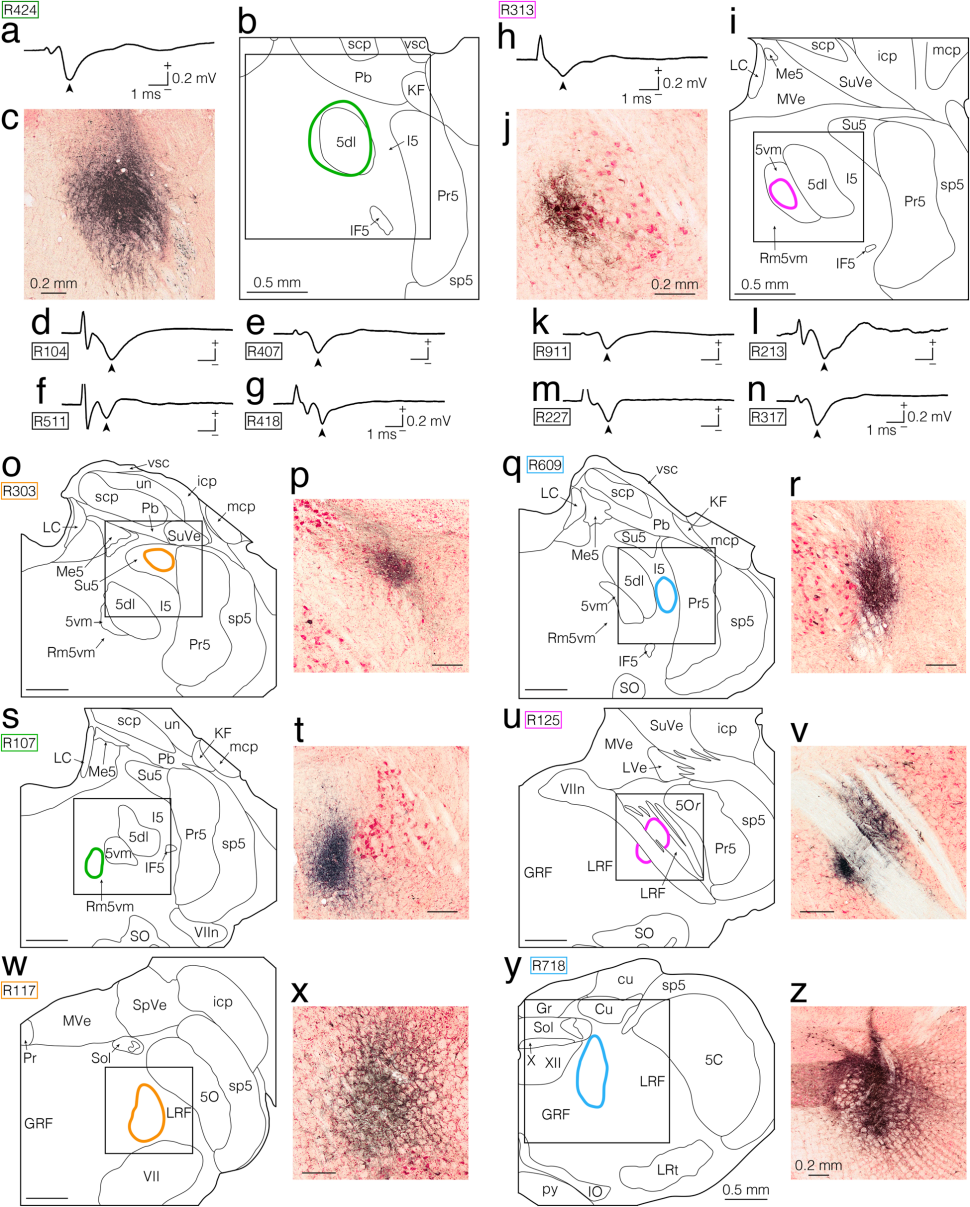
Electrophysiologically evoked responses recorded in the 5dl (**a, d–g**) and 5vm (**h, k–n**) and injection sites of retrograde tracer cholera toxin B subunit (CTb) (**b**, **c**, **i, j, o–z**) shown in coronal sections. Field potentials (**a, d–g**) and (**h, k–n**) were evoked antidromically by electrical stimulation of the ipsilateral masseter nerve in rats R424, R104, R407, R511 and R418, and the ipsilateral mylohyoid nerve in rats R313, R911, R213, R227 and R317, respectively. Arrowheads indicate peak responses with approximate latencies of 1.3 ms (**a**), 1.7 ms (**d**), 1.5 ms (**e**), 1.4 ms (**f**), 1.7 ms (**g**), 1.4 ms (**h**), 1.5 ms (**k**), 1.6 ms (**l**), 1.7 ms (**m**), 1.3 ms (**n**). CTb was injected into the 5dl in rat R424 (**b**, **c**), 5vm in rat R313 (**i**, **j**), Su5 in rat R303 (**o**, **p**), I5 in rat R609 (**q**, **r**), Rm5vm in rat R107 (**s**, **t**), LRF medial to the 5Or in rat R125 (**u**, **v**), LRF in the caudal pons in rat R117 (**w**, **x**), and dorsolateral GRF in the rostral medulla in rat R718 (**y**, **z**) on the right side. Boxed areas in (**b**, **i**, **o**, **q**, **s**, **u**, **w,** and **y**) correspond respectively to the areas taken in photomicrographs (**c**, **j**, **p**, **r**, **t**, **v**, **x,** and **z**). For abbreviations, see the abbreviations list. Scale bars = 0.1 ms and 0.2 mV in (**d**)-(**f**) and (**k**)-(**m**) as in (**g**) and (**n**); 0.5 mm in (**o**), (**q**), (**s**), (**u**) and (**w**) as in (**y**); 0.2 mm in (**p**), (**r**), (**t**), (**v**) and (**x**) as in (**z**)

On the contralateral (left) side, many labeled fibers joining the un extended rostrolaterally and surrounded dorsolaterally the vestibulocerebellar nucleus (CbVe), scp, and superior vestibular nucleus (SuVe) (Fig. 3c), and then turned ventrally (Fig. 3b, c). More caudally, many labeled axon fibers in the un extended caudo-ventromedially into the inferior cerebellar peduncle (icp), lateral vestibular nucleus (LVe) and reticular formation between the LVe and the 5Or and between the LVe or medial vestibular nucleus (MVe) and the 5O (Fig. 3c, d). Some axon terminals were labeled in the 5Or and LVe (Fig. 3c, d). Many labeled axon terminals were also seen in the dorsal LRF medial to the 5Or (Figs. 3c, 4j), and the dorsal LRF (Figs. 3d, 4k) and dorsolateral GRF (Fig. 3d, e) in the caudal pons. However, a few labeled terminals were also seen in the 5O, but none in the VII (Fig. 3d, e). More caudally at the medullary level, many axon fibers descended in the dorsolateral GRF and terminated there (Figs. 3f, g, 4l). Some labeled terminals were seen in the lateral part of the spinal vestibular nucleus (SpVe) (Fig. 3e), and were aggregated in the parasolitary nucleus (PaSol) (Fig. 3f) and IO (Fig. 3g). However, no terminals were labeled in the 5I, 5C, LRt, Amb and XII (Fig. 3f, g). In addition, many labeled axon fibers passing through the above-mentioned reticular formation between the 5Or and the LVe (Fig. 3c) ascended and terminated in the I5 (Fig. 3a, b). Labeled terminals were also seen in the Rm5vm (Figs. 3a, b, 4i), 5vm, 5dl (Figs. 3a, b, 4h), and Su5 (Figs. 3a, b, 4g), but none in the Pr5 (Fig. 3a, b). Furthermore, the above-mentioned labeled fibers in the un (Fig. 3b, c) ascended more rostrally, entered the scp (Fig. 3a), and then extended into the mesencephalic reticular formation dorsal to the scp and lateral to the decussation of the scp (not shown in Fig. 3). Finally, they ascended towards the midbrain.

On the right side (ipsilateral to the BDA injection site), the rostro-ventrolaterally extending labeled fibers arising from the BDA injection site in the jcms-MedDL traveled laterally through the LVe towards the ipsilateral icp at the rostrocaudal mid-level of the pons (Fig. 3d), and ascended in the icp which possibly includes the juxtarestiform body (Fig. 3b-d). Labeled axon terminals were found in the LVe (Fig. 3d). At the rostral pontine level, labeled axon fibers left the icp and extended ventrally to course in the I5 (Fig. 3a, b). Labeled axon terminals were very few in the I5, and none were apparent in the 5dl, 5vm, Rm5vm, 5Or and Pr5 (Fig. 3a, b). On the other hand, some labeled fibers described above in the icp at the rostrocaudal mid-level of the pons left the icp to extend ventromedially in the reticular formation between the LVe and the 5O (Fig. 3d) and terminate in the dorsal LRF ventral to the vestibular nuclei (Fig. 3d) and in the MVe at the caudal pontine level (Fig. 3e). A few axon terminals were labeled in the 5O, but none in the VII (Fig. 3d, e). At the medullary level, some descending labeled axon fibers were seen in the icp. A few labeled terminals were found in the lateral part of rostrocaudally middle LRt (Fig. 3g), but none in the 5I, 5C, Amb, and XII (Fig. 3f, g).

In summary, the labeled axon fibers and terminals in the pons and medulla arising from the jcms-MedDL showed a remarkable contralateral predominance, and so there was very little overlap of the terminals labeled from the left and right jcms-MedDL (Fig. 3). Taken together with the findings for the jcms-IntDL (Fig. 2), the laterality of the predominant projections to the pons and medulla from the jcms-MedDL was opposite to that of the jcms-IntDL, and the labeled axon fibers and terminals of the jcms-IntDL were also distributed more medially than those of the jcms-MedDL on the contralateral side. Therefore, there was little overlap of the labeled axon terminals from the jcms-IntDL and jcms-MedDL on the same side.

### Retrogradely Labeled Neurons in the Cerebellar Nuclei after CTb Injections into the Pontine and Medullary Projection Sites of the jcms-IntDL and jcms-MedDL

**Fig. 6:**
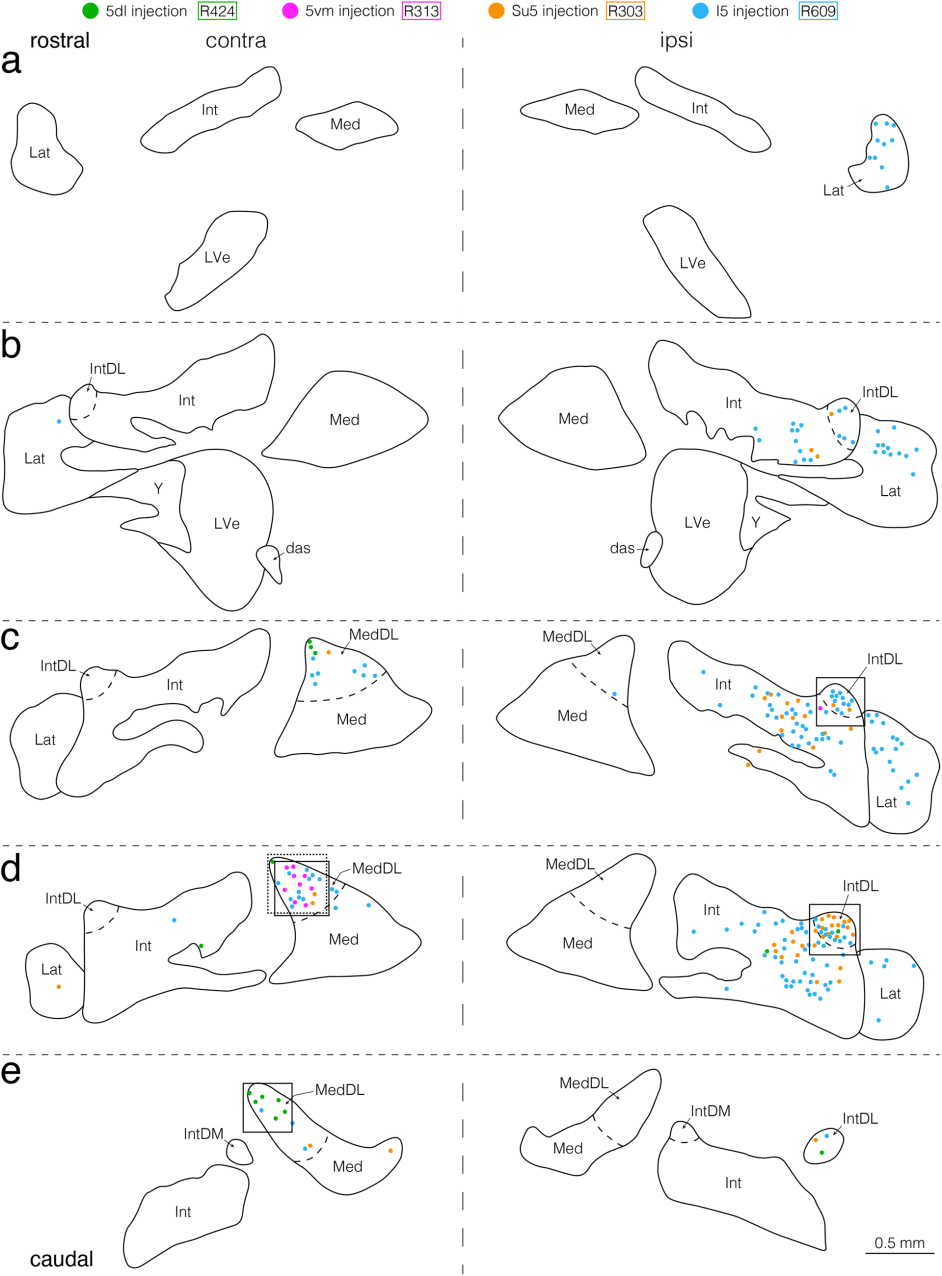
Semi-schematic drawings showing the distribution of retrogradely labeled neuronal cell bodies in the bilateral cerebellar nuclei after CTb injections into the pontine regions, where anterogradely labeled axon terminals from the jcms-IntDL or jcms-MedDL were observed in the first experiment. **a–e** Drawings of five coronal sections arranged rostrocaudally from (**a**) to (**e**). Panels (**a–e**) show the combined data from four rats R424, R313, R303, and R609. CTb injection sites in the 5dl in rat R424, 5vm in rat R313, Su5 in rat R303, and I5 in rat R609 are shown in Fig. 5b, c, Fig. 5i, j, Fig. 5o, p, and Fig. 5q, r, respectively. The left and right sides of panels (**a–e**) correspond respectively to the sides contralateral and ipsilateral to CTb injection sites. Cell bodies labeled after CTb injections into the 5dl, 5vm, Su5, and I5 are denoted by green, pink, yellow, and blue dots, respectively. Boxed areas with solid lines in (**c**) and (**d**) (left box), dotted lines in (**d**) (left box), and solid lines in (**d**) (right box) and (**e**) contain labeled cells after injections into the I5, 5vm, I5, Su5, and 5dl, respectively. The boxed area with solid lines in (**c**) corresponds to the boxed area in Fig. 7g and the region shown in Fig. 7h. The boxed area with solid lines in (**d**) (left box) corresponds to the boxed area in Fig. 7c and the region shown in Fig. 7d. The boxed area with dotted lines in (**d**) (left box) corresponds to the boxed area in Fig. 7i and the region shown in Fig. 7j. The boxed area with solid lines in (**d**) (right box) corresponds to the boxed area in Fig. 7e and the region shown in Fig. 7f. The boxed area with solid lines in (**e**) corresponds to the boxed area in Fig. 7a and the region shown in Fig. 7b. For abbreviations, see the abbreviations list. Scale bar = 0.5 mm in (**e**) (also applies to (**a**)-(**d**))

**Fig. 7:**
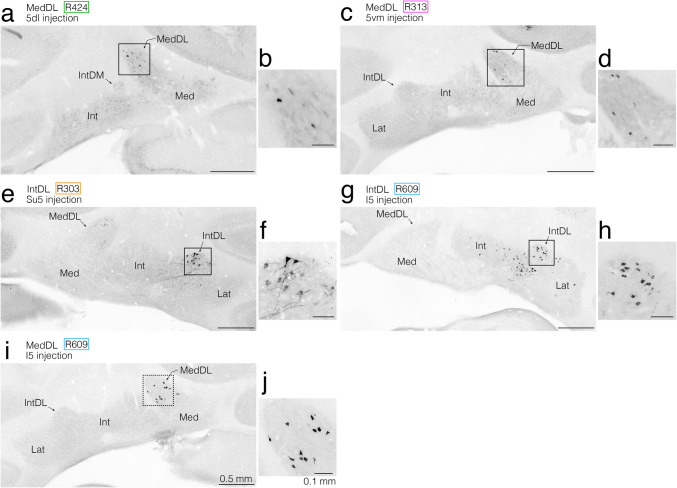
Photomicrographs showing retrogradely labeled neuronal cell bodies in the cerebellar nuclei after CTb injections into the pontine regions, where anterogradely labeled axon terminals from the jcms-IntDL or jcms-MedDL were observed in the first experiment. **a, b** Labeled neuronal cell bodies in the cerebellar nuclei (focusing on the MedDL) contralateral to the CTb injection site in the 5dl in rat R424. The boxed area in (**a**) corresponds to the region shown in (**b**) and to the boxed area with solid lines in Fig. 6e. **c, d** Labeled neuronal cell bodies in the cerebellar nuclei (focusing on the MedDL) contralateral to the CTb injection site in the 5vm in rat R313. The boxed area in (**c**) corresponds to the region shown in (**d**) and to the boxed area with solid lines in Fig. 6d (left box). **e, f** Labeled neuronal cell bodies in the cerebellar nuclei (focusing on the IntDL) ipsilateral to the CTb injection site in the Su5 in rat R303. The boxed area in (**e**) corresponds to the region shown in (**f**) and to the boxed area with solid lines in Fig. 6d (right box). **g, h** Labeled neuronal cell bodies in the cerebellar nuclei (focusing on the IntDL) ipsilateral to the CTb injection site in the I5 in rat R609. The boxed area in (**g**) corresponds to the region shown in (**h**) and to the boxed area with solid lines in Fig. 6c. **i, j** Labeled neuronal cell bodies in the cerebellar nuclei (focusing on the MedDL) contralateral to the CTb injection site in the I5 in rat R609. The boxed area in (**i**) corresponds to the region shown in (**j**) and to the boxed area with dotted lines in Fig. 6d (left box). For abbreviations, see the abbreviations list. Scale bars = 0.5 mm in (**a**), (**c**), (**e**), and (**g**) as in (**i**); 0.1 mm in (**b**), (**d**), (**f**), and (**h**) as in (**j**)

**Fig. 8:**
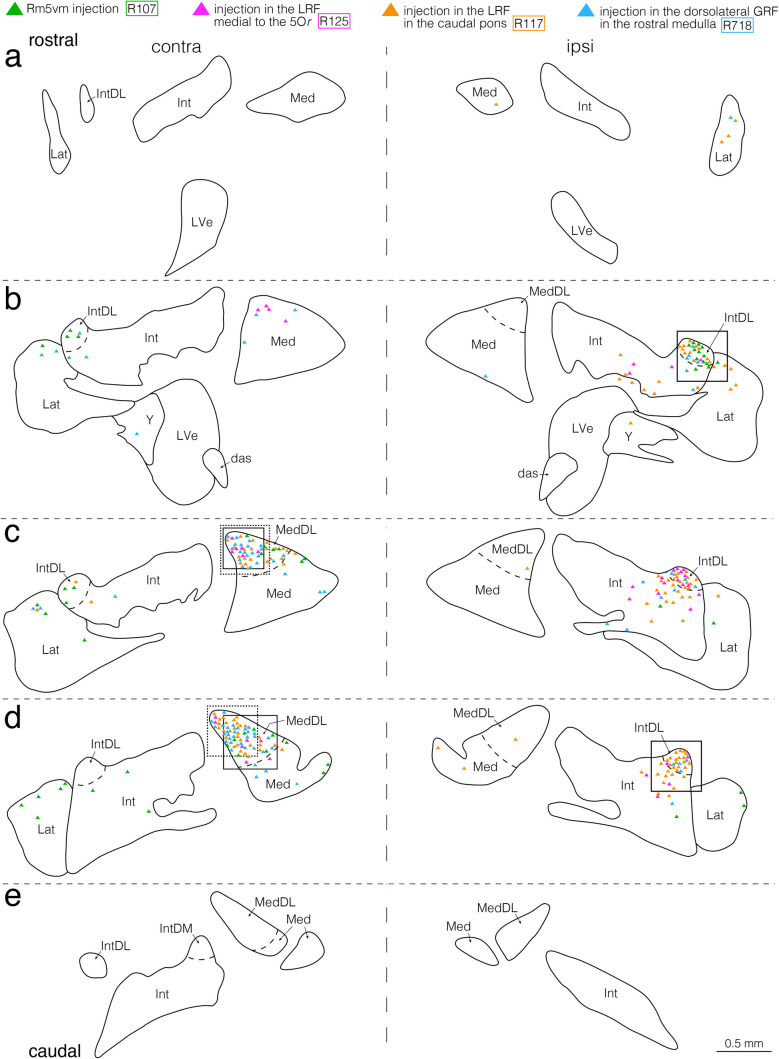
Semi-schematic drawings showing the distribution of retrogradely labeled neuronal cell bodies in the bilateral cerebellar nuclei after CTb injections into the pontomedullary regions, where anterogradely labeled axon terminals from the jcms-IntDL or jcms-MedDL were seen in the first experiment. **a–e** Drawings of five coronal sections arranged rostrocaudally from (**a**) to (**e**). Panels (**a–e**)﻿ show the combined data from four rats, R107, R125, R117, and R718. CTb injection sites in the Rm5vm in rat R107, LRF medial to the 5Or in rat R125, LRF in the caudal pons in rat R117, and dorsolateral GRF in the rostral medulla in rat R718 are shown in Fig. 5s, t, Fig. 5u, v, Fig. 5w, x, and Fig. 5y, z, respectively. The left and right sides of panels (**a–e**) correspond respectively to the sides contralateral and ipsilateral to CTb injection sites. Cell bodies labeled after CTb injections into the Rm5vm, LRF medial to the 5Or, LRF in the caudal pons, and dorsolateral GRF in the rostral medulla are denoted by green, pink, yellow, and blue triangles, respectively. Boxed areas with solid lines in (**b**) and (**c**), dotted lines in (**c**), solid lines in (**d**) (left box), dotted lines in (**d**) (left box) and solid lines in (**d**) (right box) contain labeled cells after injections into the Rm5vm, LRF medial to the 5Or, LRF in the caudal pons, Rm5vm, dorsolateral GRF in the rostral medulla, and LRF in the caudal pons, respectively. The boxed area with solid lines in (**b**) corresponds to the boxed area in Fig. 9a and the region shown in Fig. 9b. The boxed area with solid lines in (**c**) corresponds to the boxed area in Fig. 9e and the region shown in Fig. 9f. The boxed area with dotted lines in (**c**) corresponds to the boxed area in Fig. 9g and the region shown in Fig. 9h. The boxed area with solid lines in (**d**) (left box) corresponds to the boxed area in Fig. 9c and the region shown in Fig. 9d. The boxed area with dotted lines in (**d**) (left box) corresponds to the boxed area in Fig. 9k and the region shown in Fig. 9l. The boxed area with solid lines in (**d**) (right box) corresponds to the boxed area in Fig. 9i and the region shown in Fig. 9j. For abbreviations, see the abbreviations list. Scale bar = 0.5 mm in (**e**) (also applies to (**a**)-(**d**))

**Fig. 9:**
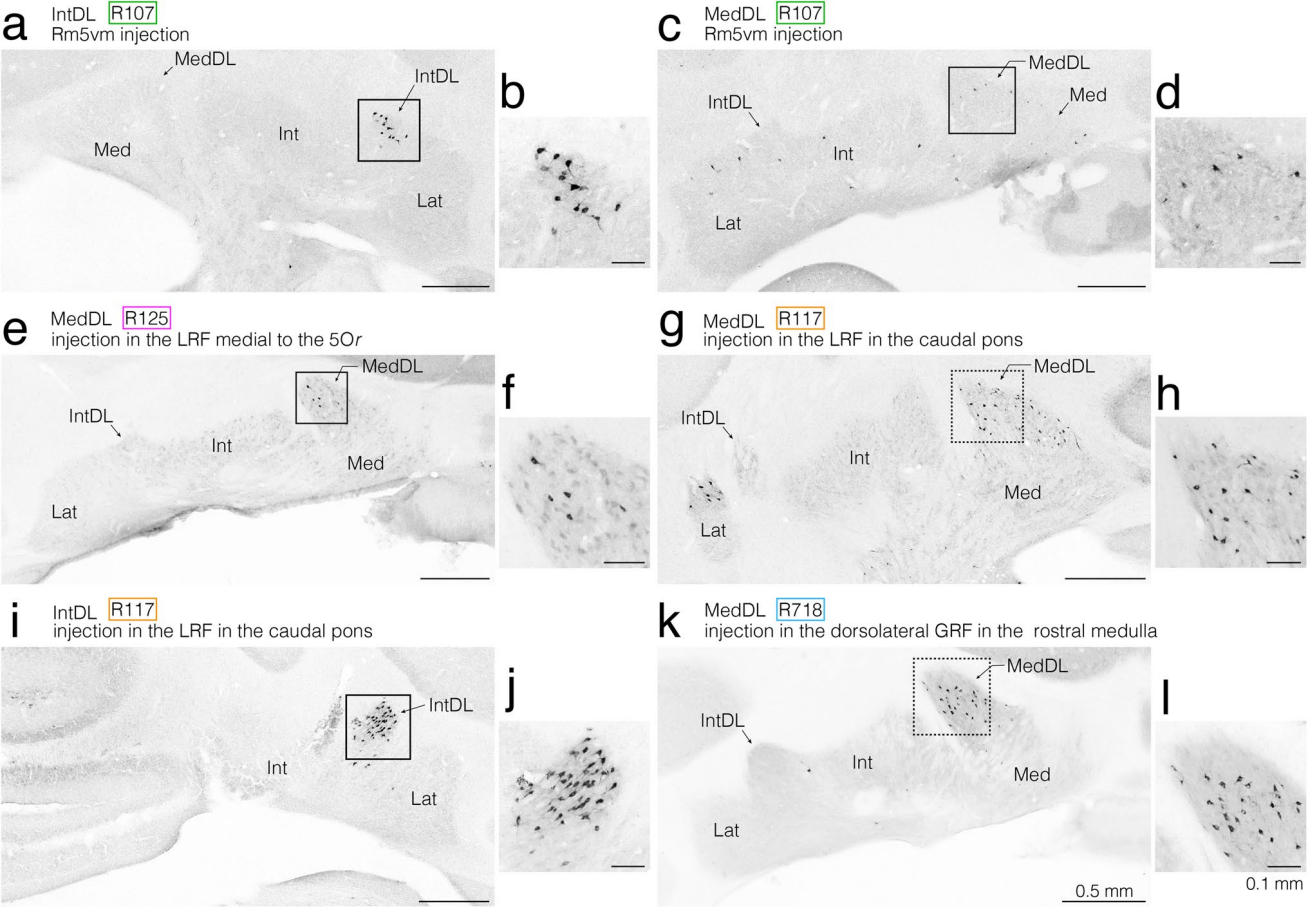
Photomicrographs showing retrogradely labeled neuronal cell bodies in the cerebellar nuclei after CTb injections into the pontomedullary regions, where anterogradely labeled axon terminals from the jcms-IntDL or jcms-MedDL were observed in the first experiment. **a, b** Labeled neuronal cell bodies in the cerebellar nuclei (focusing on the IntDL) ipsilateral to the CTb injection site in the Rm5vm in rat R107. The boxed area in (**a**) corresponds to the region shown in (**b**) and to the boxed area with solid lines in Fig. 8b. **c, d** Labeled neuronal cell bodies in the cerebellar nuclei (focusing on the MedDL) contralateral to the CTb injection site in the Rm5vm in rat R107. The boxed area in (**c**) corresponds to the region shown in (**d**) and to the boxed area with solid lines in Fig. 8d (left box). **e, f** Labeled neuronal cell bodies in the cerebellar nuclei (focusing on the MedDL) contralateral to the CTb injection site in the LRF medial to the 5Or in rat R125. The boxed area in (**e**) corresponds to the region shown in (**f**) and to the boxed area with solid lines in Fig. 8c. **g, h** Labeled neuronal cell bodies in the cerebellar nuclei (focusing on the MedDL) contralateral to the CTb injection site in the LRF in the caudal pons in rat R117. The boxed area in (**g**) corresponds to the region shown in (**h**) and to the boxed area with dotted lines in Fig. 8c. **i, j** Labeled neuronal cell bodies in the cerebellar nuclei (focusing on the IntDL) ipsilateral to the CTb injection site in the LRF in the caudal pons in rat R117. The boxed area in (**i**) corresponds to the region shown in (**j**) and to the boxed area with solid lines in Fig. 8d (right box). **k, l** Labeled neuronal cell bodies in the cerebellar nuclei (focusing on the MedDL) ipsilateral to the CTb injection site in the dorsolateral GRF in the rostral medulla in rat R718. The boxed area in (**k**) corresponds to the region shown in (**l**) and to the boxed area with dotted lines in Fig. 8d. For abbreviations, see the abbreviations list. Scale bars = 0.5 mm in (**a**), (**c**), (**e**), (**g**) and (**i**) as in (**k**); 0.1 mm in (**b**), (**d**), (**f**), (**h**) and (**j**) as in (**l**)

In the second experiment, injections of the retrograde tracer CTb were made into the pontomedullary areas where anterogradely BDA-labeled axon terminals were mainly found as described above to examine the distribution of projection neurons within the entire cerebellar nuclei (e.g., Figs. 5–9). For all 32 animals studied in the second experiment, Table 2 outlines the center and extent of the CTb deposits injected into each of the pontomedullary areas, and representative examples are shown in Fig. 5. Injections were made into the 5dl (Fig. 5b, c), 5vm (Fig. 5i, j), Su5 (Fig. 5o, p), 5I (Fig. 5q, r), Rm5vm (Fig. 5s, t), LRF medial to the 5Or (Fig. 5u, v), LRF in the caudal pons (Fig. 5w, x), and dorsolateral GRF in the rostral medulla (Fig. 5y, z) on the right side. For CTb injections into the 5dl (that included jaw-closing motoneurons), the 5dl was located in five rats by recording large, short-latency antidromic field potentials evoked by electrical stimulation of the masseter nerve, as previously described [35, 43]. The peak response latencies recorded in the 5dl were approximately 1.3 ms in rat R424 (a), 1.7 ms in rat R104 (d), 1.5 ms in rat R407 (e), 1.4 ms in rat R511 (f), and 1.7 ms in rat R418 (g). The CTb deposits in all five rats were almost entirely confined to the 5dl on the right side; the CTb deposit in a representative rat R424 is shown in Fig. 5b, c and the resultant labeled cerebellar nucleus neurons were found to be limited in the location, being confined almost entirely in the contralateral caudal MedDL (Figs. 6 and 7a, b). For CTb injections into the 5vm (that included jaw-opening motoneurons), the 5vm was located by recording antidromic responses after electrical stimulation of the mylohyoid nerve as previously described [35, 43]. The peak response latencies recorded in the 5vm were approximately 1.4 ms in rat R313 (h), 1.5 ms in rat R911 (k), 1.6 ms in rat R213 (l), 1.7 ms in rat R227 (m), and 1.3 ms in rat R317 (n). The CTb deposits in all five rats were well confined to the 5vm on the right side; the CTb deposit in a representative rat R313 is shown in Fig. 5i, j and the resultant labeled cerebellar nucleus neurons were largely confined to the contralateral caudal MedDL (Figs. 6, 7c, d). The other targets for CTb injections—the Su5, I5, Rm5vm, LRF medial to the 5Or, and dorsolateral GRF in the rostral medulla—were located on the right side as described in the Materials and Methods. The resultant extents of CTb-deposits were confined to the Su5, I5, Rm5vm, LRF medial to the 5Or, and dorsolateral GRF in the rostral medulla in three rats each (Table 2). Findings from a representative rat R303 where the CTb injection was confined to the Su5 (Fig. 5o, p) showed many labeled neurons ipsilaterally in the IntDL and dorsolateral Int adjacent to the IntDL and fewer were observed contralaterally in the MedDL (Figs. 6, 7e, f). In a representative rat R609, the CTb deposit was confined to the I5 (Fig. 5q, r), and many neurons were labeled ipsilaterally in the IntDL (Fig. 6), Int adjacent to the IntDL (Figs. 6, 7g, h) and lateral cerebellar nucleus (Lat) (Fig. 6) and contralaterally in the MedDL (Figs. 6, 7i, j). Findings in a representative rat R107 where the injection site was located in the Rm5vm (Fig. 5s, t) revealed labeled neurons ipsilaterally in the IntDL (Fig. 8, 9a, b), and fewer labeled neurons contralaterally in the IntDL, MedDL (Figs. 8, 9c, d) and Lat (Fig. 8). In a representative rat R125, the CTb deposit was seen in the LRF medial to the 5Or (Fig. 5u, v), and many neurons were labeled contralaterally in the rostral MedDL (Figs. 8, 9e, f), and fewer neurons ipsilaterally in the caudal IntDL (Fig. 8). In a representative rat R117 showing the CTb injection site in the LRF in the caudal pons (Fig. 5w, x), many neurons were labeled contralaterally in the rostral MedDL (Figs. 8, 9g, h) and ipsilaterally in the IntDL (Fig. 8) and Int adjacent to the IntDL (Fig. 9i, j). In a representative rat R718 where the CTb deposit was located mainly in the dorsolateral GRF but extended slightly into the dorsomedial LRF in the rostral medulla (Fig. 5y, z), many neurons were labeled contralaterally in the rostral MedDL (Figs. 8, 9k, l), and fewer were labeled ipsilaterally in the IntDL (Fig. 8).

In summary, the distribution of retrogradely CTb-labeled cerebellar nuclear neurons revealed that amongst the entire cerebellar nuclei most neurons projecting to the 5dl, 5vm, Su5, 5I, Rm5vm, LRF medial to the 5Or, LRF in the caudal pons, and dorsolateral GRF in the rostral medulla were labeled in the IntDL and MedDL, and that the IntDL and MedDL projected to distinct pontomedullary regions.

### Retrogradely Labeled Premotoneurons after CTb Injections into the 5dl and 5vm

In the third experiment, we examined the distribution of retrogradely labeled premotoneurons projecting to the 5dl (premotoneurons for the 5dl) and the 5vm (premotoneurons for the 5vm) in the pons and medulla in cases where neurons in the cerebellar nuclei were retrogradely labeled after CTb injections into the 5dl and 5vm had been examined in the second experiment (e.g., Fig. 5b, c, i, j) (Table 2).

#### Premotoneurons for the 5dl

**Fig. 10:**
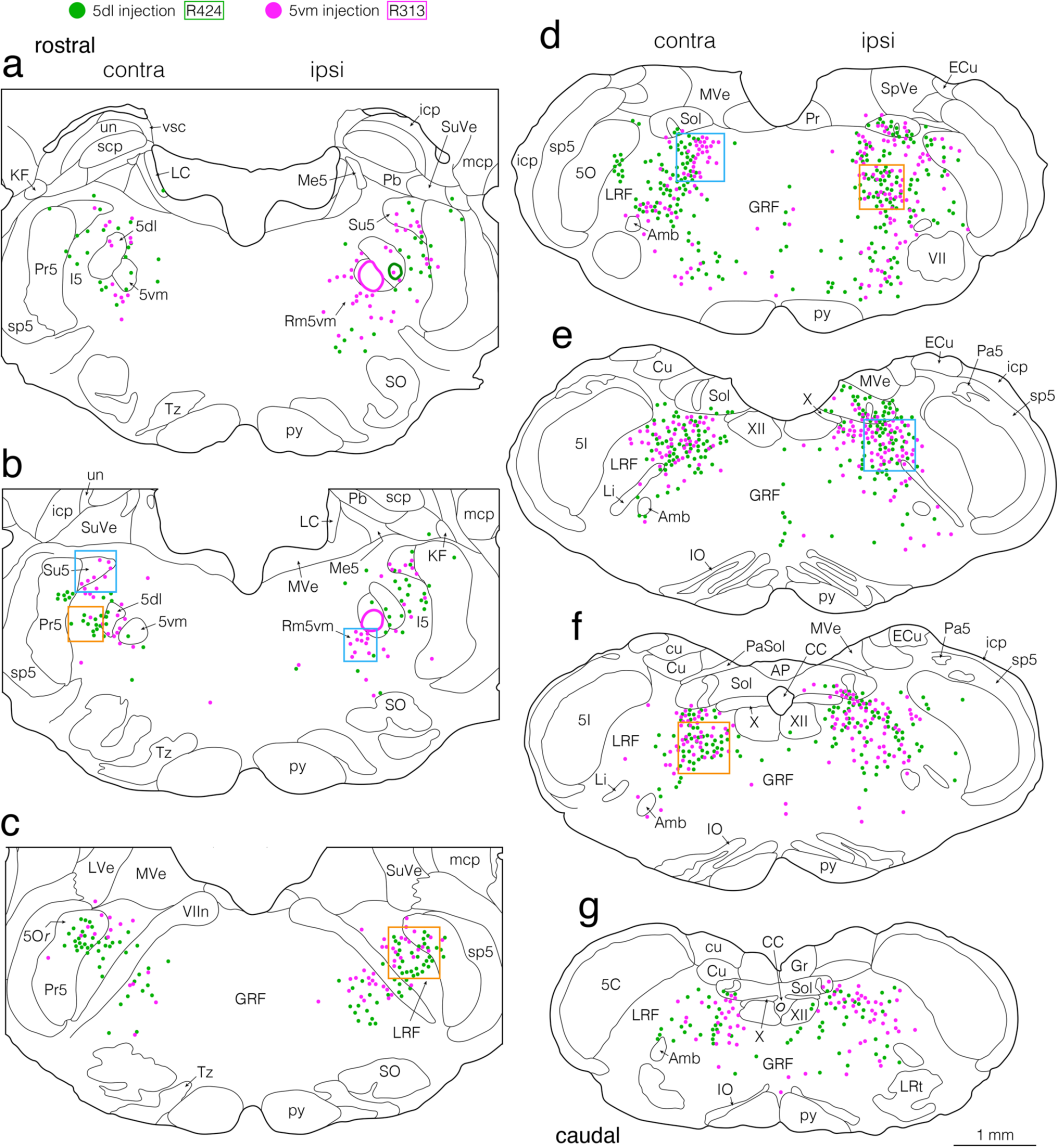
Semi-schematic drawings showing the distribution of retrogradely labeled neuronal cell bodies (denoted by green and pink dots) after CTb injections into the 5dl in rat R424 and into the 5vm in rat R313, respectively. Seven coronal sections (**a–g**) are rostrocaudally arranged from (**a**) to (**g**). Left and right sides of panels (**a–g**) correspond to the sides contralateral and ipsilateral to CTb injection sites, respectively. The centers of injection sites and recordings from the injection sites in rats R424 and R313 are presented in Fig. 5a–c and Fig. 5h–j, respectively. Since the injection center in the 5dl was located rostral to the level shown in (**a**), the caudal extent of the injection deposit is denoted by a green circle in (**a**). On the other hand, since the injection center in the 5vm was located only a little rostral to the level shown in (**a**), the caudal extent of the injection deposit is denoted by pink circles in (**a** and **b**). Boxed areas with yellow lines in (**b**), (**c**), (**d**), and (**f**) contain neurons labeled after a 5dl injection in rat R424, in the contralateral I5, ipsilateral LRF medial to the 5Or, ipsilateral LRF in the caudal pons, and contralateral GRF in the rostral medulla, respectively, and correspond to the areas in photomicrographs in Fig. 11a–d, respectively. Boxed areas with blue lines in (**b**) (left box), (**b**) (right box), (**d**) and (**e**) include neurons labeled after a 5vm injection in rat R313, in the contralateral Su5, ipsilateral Rm5vm, contralateral LRF in the caudal pons, and ipsilateral GRF in the rostral medulla, respectively, and correspond to the areas in photomicrographs in Fig. 11e–h, respectively. For abbreviations, see the abbreviations list. Scale bar = 1 mm in (**g**) (also applies to (**a**)-(**f**))

**Fig. 11:**
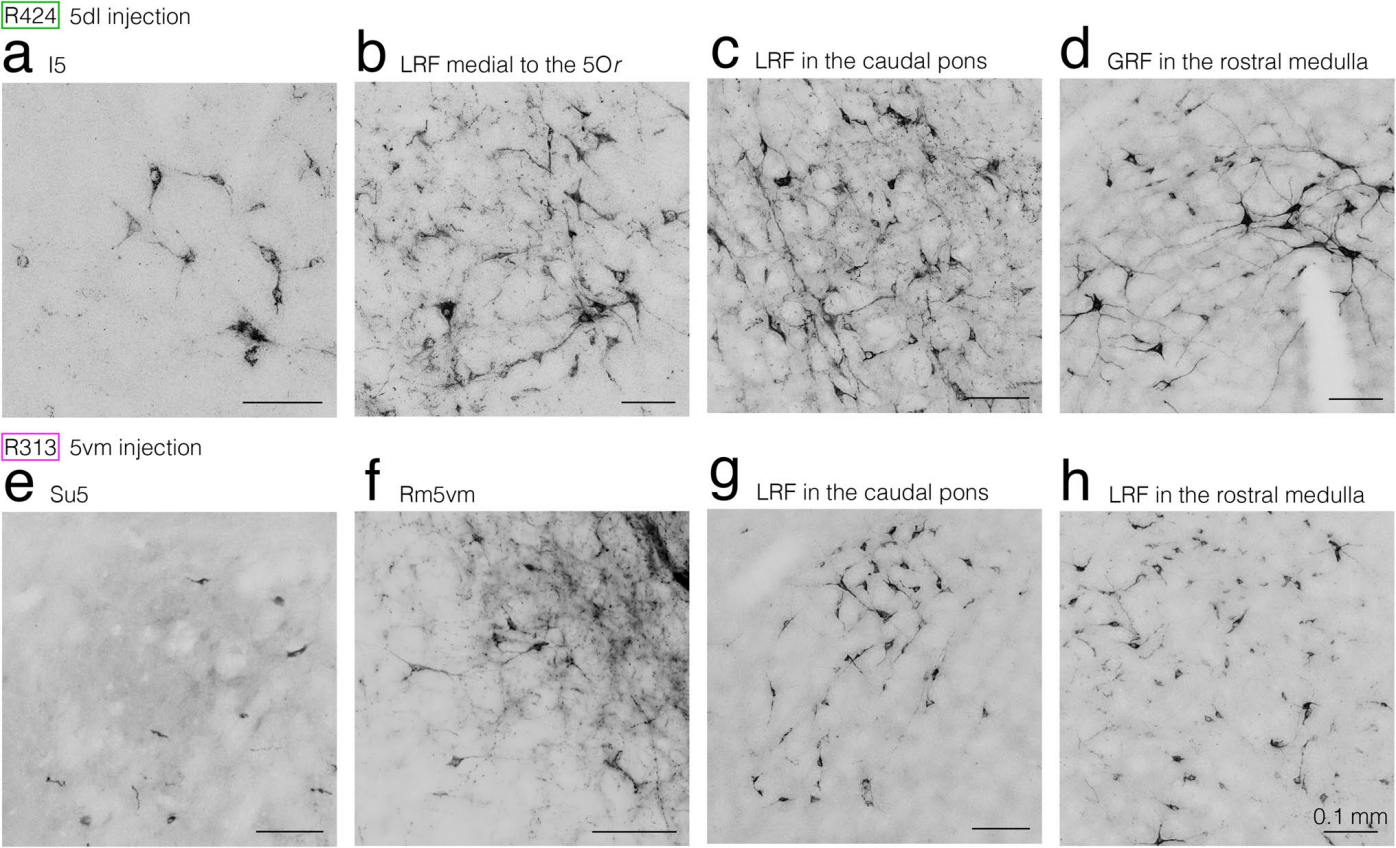
Photomicrographs showing retrogradely labeled neuronal cell bodies after CTb injections into the 5dl in rat R424 (**a–d**) and into the 5vm in rat R313 (**e–h**). Photomicrographs (**a–h**) show the cell bodies labeled in the contralateral I5, ipsilateral LRF medial to the 5Or, ipsilateral LRF in the caudal pons, and contralateral GRF in the rostral medulla, contralateral Su5, ipsilateral Rm5vm, contralateral LRF in the caudal pons, and ipsilateral LRF in the rostral medulla, respectively, and correspond to boxed areas with yellow lines in (**b**), (**c**), (**d**), and (**f**), and those with blue lines in (**b**) (left box), (**b**) (right box), (**d**), and (**e**), respectively, in Fig. 10. For abbreviations, see the abbreviations list. Scale barss = 0.1 mm in (**a**)-(**g**) as in (**h**)

The CTb injection site in a representative rat R424 was confined mostly to the 5dl on the right side, as shown in Fig. 5b, c. At the rostralmost pontine level, no retrogradely labeled neurons were seen in the RtTg bilateral to the CTb injection site. At the rostral pontine levels many premotoneurons for the 5dl were labeled bilaterally in the I5 (Figs. 10a, b, 11a), 5Or and dorsolateral LRF medial to the 5Or (Figs. 10c, 11b). Fewer neurons were labeled bilaterally in the Su5 but these had a clear ipsilateral predominance (Fig. 10a, b). However, neurons were rarely labeled bilaterally in the Rm5vm and IF5. At the caudal pontine level, many premotoneurons for the 5dl were observed bilaterally in the medial LRF (Figs. 10d, 11c). Fewer neurons were labeled bilaterally in the lateral LRF medial to the 5O, and bilaterally with an ipsilateral predominance in the 5O, nucleus of the solitary tract (Sol) and ventral GRF (Fig. 10d). At the medullary level, many premotoneurons for the 5dl were seen bilaterally in the dorsomedial LRF ventrolateral to the rostral Sol (Fig. 10e, f) and dorsolateral GRF (Figs. 10f, g, 11d) and bilaterally with a clear ipsilateral predominance in the rostral Sol (Fig. 10e, f). Almost no premotoneurons for the 5dl were labeled in the Li and none were evident in the PaSol and IO.

In summary, most premotoneurons for the 5dl were found in bilaterally corresponding pontomedullary regions.

#### Premotoneurons for the 5vm

The injected CTb deposit in a representative rat R313 was mostly confined to the 5vm, as shown in Fig. 5i, j. At the rostralmost pontine level, no labeled neurons were seen bilaterally in the RtTg. At the rostral pontine level, labeled premotoneurons for the 5vm were seen bilaterally in the Su5 (Figs. 10a, b, 11e) and bilaterally with an ipsilateral predominance in the Rm5vm (Figs. 10a, b, 11f), and dorsolateral LRF medial to the 5Or. A few premotoneurons for the 5vm were also labeled bilaterally in the medial 5Or (Fig. 10c). At the caudal pontine level, many premotoneurons for the 5vm were present bilaterally in the LRF, especially in its dorsomedial region (Figs. 10d, 11g) and a few neurons were labeled ipsilaterally in the dorsal 5O (Fig. 10d). At the medullary level, many premotoneurons for the 5vm were bilaterally labeled in the dorsomedial LRF (Figs. 10e-g, 11h). Fewer labeled neurons were seen ipsilaterally in the ventral Sol (Fig. 10e-g) and bilaterally in the dorsal GRF (Fig. 10f, g).

**Fig. 12:**
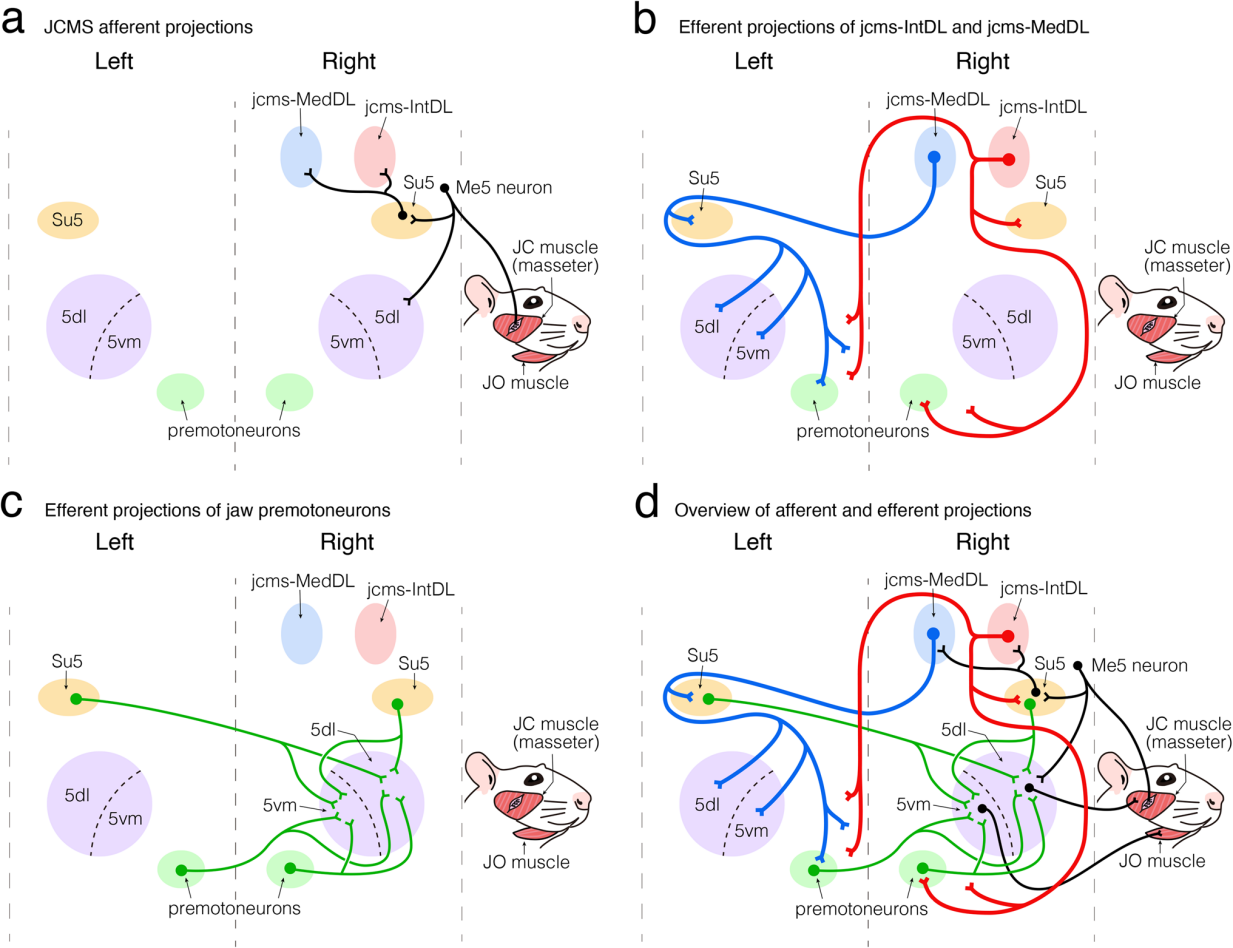
Diagram showing the projection patterns of the various neural components documented in the present study. **a–d** For simplicity, only the projections from the right side are illustrated. **a** JCMS afferent projections to the jcms-IntDL and jcms-MedDL through the trigeminal mesencephalic nucleus neurons as well as the Su5 neurons. **b** Efferent projections of the jcms-IntDL and jcms-MedDL. **c** Efferent projections of premotoneurons to the 5dl and 5vm in the Mo5. **d** The overview of afferent and efferent projections presented in (**a**, **b**, and **c**). Projections of JC motoneurons in the 5dl that innervate JC muscles and projections of jaw-opening motoneurons in the 5vm that innervate jaw-opening muscles are also presented. For abbreviations, see the abbreviations list

In summary, these findings showed that most premotoneurons for the 5vm were found in bilaterally corresponding pontomedullary regions. Taken together with the findings for the 5dl, it is apparent that premotoneurons for the 5dl and premotoneurons for the 5vm were widely distributed bilaterally in the approximately same regions in the pons and medulla (also see Fig. 12c, d).

### Overlap Between the Distribution of Axon Terminals from the jcms-IntDL and jcms-MedDL and the Distribution of Premotoneurons for the 5dl or 5vm

We analyzed the overlap between the distribution of anterogradely labeled axon terminals from the jcms-IntDL or jcms-MedDL (Figs. 2 and 3) and the distribution of retrogradely labeled premotoneurons for the 5dl or 5vm (Fig. 10) to reveal possible indirect pathways from the jcms-IntDL or jcms-MedDL to the 5dl and 5vm.

#### Terminals from the jcms-IntDL and Premotoneurons

Overlap was predominantly seen on the side ipsilateral to the jcms-IntDL (also see Fig. 12d). For instance, a strong overlap was found in the ipsilateral LRF medial to the 5Or, where many premotoneurons for the ipsilateral 5dl, ipsilateral 5vm, and contralateral 5dl were found (Fig. 2c and Fig. 10c). Overlap was also observed in the ipsilateral LRF medial to the caudal 5I, where premotoneurons for the ipsilateral 5dl and ipsilateral 5vm were seen (Fig. 2f and Fig. 10f), and in the ipsilateral I5, where premotoneurons for the ipsilateral 5dl and contralateral 5dl were seen (Fig. 2a, b and Fig. 10a, b). On the side contralateral to the jcms-IntDL, only a weak overlap was present in the Rm5vm, where premotoneurons for the ipsilateral 5vm were observed.

In summary, these findings suggest that the jcms-IntDL on one side may send indirect projections to bilateral 5dl and 5vm via several premotoneurons in the pontomedullary regions.

#### Terminals from the jcms-MedDL and Premotoneurons

Overlap was predominantly seen on the side contralateral to the jcms-MedDL (Fig. 12d). For example, a strong overlap was found in the contralateral LRF medial to the 5Or, where there were many premotoneurons for the contralateral 5dl, ipsilateral 5dl and ipsilateral 5vm, and fewer premotoneurons for the contralateral 5vm (Fig. 3c and Fig. 10c). A considerable overlap was also present in the contralateral LRF in the caudal pons, where many premotoneurons for the contralateral 5dl and ipsilateral 5dl and fewer premotoneurons for the ipsilateral 5vm were observed (Fig. 3d and Fig. 10d, c). Overlap was also observed in the dorsal part of the contralateral GRF ventrolateral to the contralateral XII, where premotoneurons for the contralateral 5dl, ipsilateral 5dl, contralateral 5vm and ipsilateral 5vm were evident (Fig. 3e-g and Fig. 10e-g). A weak overlap was also seen in the contralateral Rm5vm, where fewer premotoneurons for the ipsilateral 5vm were found.

In summary, these findings suggest that the jcms-MedDL on one side can indirectly project bilaterally to the 5dl and 5vm via several premotoneurons in the pontomedullary regions, which were different from those described above for the jcms-IntDL.

## Discussion

To clarify the neural circuitry underlying the cerebellar control of jaw muscle function, we electrophysiologically identified the regions of the cerebellar nuclei which contained neurons receiving JCMS proprioceptive afferent inputs (i.e. jcms-IntDL and jcms-MedDL) and used both anterograde and retrograde tract tracing techniques to detail morphological features of projections from the jcms-IntDL and jcms-MedDL to the pons and medulla which include the Mo5 (5dl and 5vm) and their premotoneurons. In addition, we labeled premotoneurons for the ipsilateral 5dl or 5vm or contralateral 5dl or 5vm in the pons and medulla, and thus documented the indirect projections from the jcms-IntDL and jcms-MedDL to the 5dl and 5vm via these premotoneurons. The following subsections discuss these findings with a focus on the significance of the cerebellofugal projections from the jcms-IntDL and jcms-MedDL in the control of jaw muscle function. Figure 12 shows the projection patterns of the various neural components documented in the present study that contribute to cerebellar sensorimotor control of jaw muscle function, illustrating in turn the afferent projections of JCMS (Fig. 12a), efferent projections of the jcms-IntDL and jcms-MedDL (Fig. 12b), efferent projections of premotoneurons for the Mo5 (Fig. 12c), and the overview of these projection patterns (Fig. 12d). For simplicity, only the projections from the right side are illustrated in Fig. 12.

### Projections from the jcms-IntDL and jcms-MedDL

Our study revealed the detailed distribution of cerebellofugal projection sites in the pons and medulla originating from the rat jcms-IntDL and jcms-MedDL. The IntDL and MedDL are cytoarchitectonically prominent, respectively, in the Int and Med of rodents, but not in higher animal species [27–30]. Therefore, in monkeys [48, 49], cats [50], and dogs [51], the distribution of cerebellofugal projection sites in the pons and medulla originating only from the fastigial cerebellar nucleus (Med) has been well documented, while that from the Int has not been detailed. Teune et al. [34] have clearly shown the efferent projection sites from the cerebellar nuclei and that they are very different depending on the individual subnuclei of these nuclei, and have demonstrated the segregated efferent projection features from several subnuclei including the IntDL and MedDL in rats. However, since Teune et al. [34] did not include techniques to identify any subnuclei receiving muscle proprioceptive afferent inputs, our discussion can only include a general comparison of our findings of efferent projections with those of Teune et al. [34]. The following discussion focusses on the projections to pontomedullary regions other than the Mo5 which is considered in a separate subsection further below.

#### From the IntDL

Projections from the jcms-IntDL in our study showed a clear ipsilateral predominance similar to those from the IntDL described by Teune et al. [34]. Both studies revealed ipsilateral projections to the I5, 5O, and LRF medial to the trigeminal spinal nuclei (5O, 5I, and 5C). Our study additionally disclosed considerable ipsilateral projections to the Su5, IF5, and Li, and weak projections to the VII, and LRt, which Teune et al. [34] did not report. On the other hand, we could not find ipsilateral projections to the IO, Pb, SpVe, and Pr5 and 5C in the trigeminal sensory nuclei, which Teune et al. [34] reported. On the contralateral side, we observed projections to the pontomedullary reticular formation as well as the RtTg and IO, whereas Teune et al. [34] did not report contralateral projections to the pontomedullary reticular formation.

#### From the MedDL

Both our study and that of Teune et al. [34] showed that the jcms-MedDL and MedDL projected to the pontomedullary structures with a clear contralateral predominance. Both studies revealed contralateral projections to the LRF, 5O, SpVe, PaSol, and IO. Our study additionally showed significant projections to the 5I and weak projections to the 5vm, 5dl, Su5, and Rm5vm, which Teune et al. [34] did not report. In contrast, we did not detect contralateral projections to the RtTg, Pb, Pr5, 5I, SuVe, and SpVe, which they reported. On the ipsilateral side, we found minor projections to the pontomedullary reticular formation, LVe, and LRt, while Teune et al. [34] reported projections to the Pb, SuVe, and MVe.

We used iontophoretic injection of anterograde tracer BDA in young adult male rats, whereas Teune et al. [34] used iontophoretic injection of anterograde tracer *Phaseolus vulgaris* leucoagglutin in adult male rats. Therefore, we cannot completely exclude the possibility that these differences between the findings of the present study and those of Teune et al. [34] were due to different anterograde tracers used and also to the extent of the injection sites. For example, the injection sites made by Teune et al. [34] might not have covered the entire jcms-IntDL and jcms-MedDL, and might have included other cerebellar subnuclei beyond the jcms-IntDL and jcms-MedDL.

### Characteristics of Projections from the jcms-IntDL and jcms-MedDL

As summarized in Fig. 12b, the findings of the distributions of anterogradely BDA-labeled terminals revealed that the principal axonal projections from the jcms-IntDL and jcms-MedDL to the pons and medulla were predominantly to the ipsilateral side and contralateral side, respectively. This laterality is supported not only by Teune et al. [34] as discussed above but also by other earlier findings of cerebellofugal projections; in monkeys [48, 49], cats [50], and dogs [51] that the fastigial cerebellar nucleus (Med) gives off stronger contralateral cerebellofugal projections to the pons and medulla via the uncinate fasciculus, while its ipsilateral cerebellofugal projections to these regions via the juxtarestiform body are weaker. The ipsilaterally predominant cerebellofugal projections from the Int to the pons and medulla have also been demonstrated in rats [52], although not in higher animal species. The present findings suggest that these segregated projections from the jcms-IntDL and jcms-MedDL to different pontomedullary neurons might exert different functional outcomes. In addition, the jcms-IntDL on one side and the other side projected to different regions in the pons and medulla, and the jcms-MedDL on one side and the other side also projected to different pontomedullary regions. It is noteworthy that these characteristic projection features were confirmed by the distribution of the CTb-labeled cerebellar subnucleus neurons projecting to the pons and medulla.

The distribution of the CTb-labeled neurons also showed another noteworthy result in that the pontomedullary regions where axons from the jcms-IntDL or jcms-MedDL terminated received efferent projections that were principally from the IntDL or MedDL amongst the entire cerebellar nuclei. This topographical segregation of efferent projections from these cerebellar nuclei documented in the present study is consistent with the findings of Teune et al. [34] who made small injections of anterograde tracers separately into the individual cerebellar nuclei to examine the segregated projection features from the entire cerebellar nuclei. Furthermore, efferent projections from the cerebellar nuclei to the IO are well known to be topographically segregated [53, 54]. Accordingly, the topographically segregated projections appear to be a principle that is a general characteristic for cerebellofugal projections.

Also of relevance, the segregated cerebellar projections include the well-known cerebellar cortico-nuclear projections from the Purkinje cells that are mediolaterally segregated in a parasagittal modular pattern [27]. The IntDL receives cortico-nuclear projections from the D0 zone in the hemisphere of the cerebellar cortex, whereas the MedDL receives projections from the A2 zone in the vermis of the cerebellar cortex [55, 56]. Therefore, taken together with the present findings, it is highly likely that the cortico-nucleo-bulbar projections via the jcms-IntDL and jcms-MedDL are also segregated. The hemisphere of the cerebellar cortex has been shown to be involved in the planning of actual movements, evaluation of sensory information for action, and cognitive functions, while the vermis of the cerebellar cortex is involved in the fine tuning of body and limb movements [57–59]. This raises the possibility that these cerebral cortical functions modulate the JCMS proprioceptive inputs transmitted by neurons in the jcms-IntDL and jcms-MedDL. As such, the efferent projections from the jcms-IntDL to the pontomedullary regions might be involved in the planning of actual jaw movements, evaluation of sensory information for jaw movements, and cognitive functions, whereas the efferent projections from the jcms-MedDL to them might be more involved in the fine tuning of jaw movements.

### Projections from the jcms-IntDL and jcms-MedDL to the Mo5

Muscle proprioceptive afferent inputs to the CNS modulate the activity of motoneurons in the lower brainstem or spinal cord through mono- or polysynaptic reflex arcs (e.g., [10–13]). Accordingly, proprioceptive information from muscles is likely to play a significant role in cerebellar sensorimotor modulation through direct or indirect projections arising from the cerebellar nuclei receiving muscle proprioceptive afferent inputs to motoneurons innervating these muscles. With respect to JCMS proprioceptive signals, they are conveyed by Me5 neurons to the 5dl (JC motoneuron pool) and the Su5 which includes neurons projecting directly to the 5vm (JO motoneuron pool) as well as the 5dl [35, 60–64]. In addition, Tsutsumi et al. [26] have revealed that JCMS proprioceptive signals are conveyed via the Su5 neurons to the jcms-IntDL and jcms-MedDL. Therefore, in the present study we first examined the detailed features of direct projections from the jcms-IntDL and jcms-MedDL not only to the 5dl but also to the 5vm. However, cerebellar nucleus neurons with the direct projections to the 5dl and 5vm were found to be limited mainly to the contralateral MedDL. Thus, we next examined the distribution of premotoneurons for the 5dl and 5vm to demonstrate the indirect projections from the jcms-IntDL and jcms-MedDL not only to the 5dl but also to the 5vm via their premotoneurons.

The present study documented that many premotoneurons for the 5dl or 5vm were labeled bilaterally in the pontomedullary regions, in accord with earlier findings [35, 43, 60, 65] (also see Fig. 12c, d), and many axons arising from the jcms-IntDL or jcms-MedDL terminated in these pontomedullary regions (also see Fig. 12b, d). Tsutsumi et al. [26] have demonstrated that JCMS proprioceptive signals are conveyed through the ipsilateral Su5 to the jcms-IntDL and jcms-MedDL bilaterally (Fig. 12a). Thus, it is highly likely that JCMS proprioceptive signals elicited only on one side are transmitted from left and right jcms-IntDL and jcms-MedDL bilaterally to premotoneurons that project to left and right 5dl or 5vm (Fig. 12d). Interestingly, most jaw movements involve the bilateral activation of the jaw muscles [10, 12], so that the JCMS proprioceptive signals are bilaterally elicited during these movements. Thus, the above-described complex bilateral neuronal circuits (Fig. 12d) may be activated bilaterally during these jaw movements by JCMS proprioceptive signals arising from the JC muscles on both sides. However, the present study has revealed that the projections from the jcms-IntDL and jcms-MedDL are well segregated and target predominantly the ipsilateral and contralateral pontomedullary regions, respectively, and the distribution of axon terminals from the jcms-IntDL and jcms-MedDL on the same side show very limited overlap. Therefore, the above-described complex pathways conveying JCMS proprioceptive information to the 5dl or 5vm (Fig. 12d) might be regulated in a segregated manner by the activation of efferent projections from the jcms-IntDL and jcms-MedDL.

Interestingly, the IntDL is known to control eyelid muscles through its projections to the facial nucleus (cat, [66]; rabbit, [67]; human, [68]; rat, [69]). Morcuende et al. [70] have demonstrated that although there are direct projections from the rat IntDL to the dorsolateral division of the facial nucleus, which contains motoneurons innervating the orbicularis oculi muscle, the indirect projections via the premotoneurons are more predominant. In the present study, we observed weak indirect projections from the IntDL to the ipsilateral dorsolateral division of the facial nucleus. These findings suggest that the IntDL predominantly projects indirectly to the dorsolateral division of the facial nucleus via its premotoneurons. Interestingly, Li et al. [71] have revealed that there are premotoneurons in the pons and medulla, which directly project to both the facial nucleus and Mo5. Together with the findings in the present study, the same IntDL neurons may cooperatively control jaw-movements and eyelid movements via the same premotoneurons.

### Other Functional Considerations of Pontomedullary Projections from the jcms-IntDL and jcms-MedDL

Cicirata et al. [72] electrically stimulated the rat IntDL and suggested from their findings that it is involved in regulating oral motor activity, and Katayama et al. [73] demonstrated that electrical stimulation of the Int in guinea pigs suppresses the JC masseteric reflex and facilitates the JO reflex evoked in the anterior digastric muscle. On the other hand, in cats, electrical stimulation of the Med has been reported to induce or modulate several functions including eating [74]. In view of the present findings, these stimulation-induced effects might at least partly reflect activation of the indirect projections that we have documented from the jcms-IntDL or jcms-MedDL to the 5dl and 5vm.

## Conclusions

To help clarify the neural circuitry underlying the role of the cerebellum in the sensorimotor control of jaw muscle function, the present study used anterograde and retrograde tract tracings combined with electrophysiological recordings to document the detailed features of projections to the pons and medulla from the cerebellar nuclei (the jcms-IntDL and jcms-MedDL), which contain neurons receiving JCMS proprioceptive inputs. The jcms-IntDL and jcms-MedDL were shown to project predominantly to several pontomedullary regions on the ipsilateral side and the contralateral side, respectively. The overlap between the pontomedullary areas receiving projections from the jcms-IntDL and the jcms-MedDL was limited. Amongst the entire cerebellar nuclei, the cerebellar nucleus neurons projecting to the pontomedullary regions where the projections from the jcms-IntDL or jcms-MedDL terminated were predominantly located in the IntDL or MedDL. These findings suggest that the pontomedullary projections from the jcms-IntDL and the jcms-MedDL are well segregated from each other and may also be distinct from the cerebellofugal projections from other cerebellar nuclei. Through their indirect projections to JC or JO motoneurons in the bilateral 5dl or 5vm, the jcms-IntDL and jcms-MedDL may each have a separate role in the cerebellar control of jaw muscle function.

## Data Availability

All data and materials are available upon request.

## Abbreviations

IV: Lobule IV
V: Lobule V
VI: Lobule VI
VII: Facial nucleus
VIIn: Facial nerve
X: Vagus nerve nucleus
XII: Hypoglossal nucleus
5C: Trigeminal caudal subnucleus
5dl: Dorsolateral division of the Mo5
5I: Trigeminal interpolar subnucleus
5O: Trigeminal oral subnucleus
5Or: Rostro-dorsomedial part of the 5O
5vm: Ventromedial division of the Mo5
Amb: Ambiguus nucleus
AP: Area postrema
BDA: Biotinylated dextran amine
CbVe: Vestibulocerebellar nucleus
CC: Central canal
Crus I: Crus I of the ansiform lobule
Crus II: Crus II of the ansiform lobule
CTb: Cholera toxin B subunit
Cu: Cuneate nucleus
cu: Cuneate fasciculus
das: Dorsal acoustic stria
ECu: External cuneate nucleus
GRF: Gigantocellular reticular formation
Gr: Gracile nucleus
I5: Intertrigeminal region
icp: Inferior cerebellar peduncle
IF5: Interfascicular trigeminal nucleus
Int: Interposed cerebellar nucleus
IntDL: Dorsolateral hump of the Int
IntDM: Dorsomedial crest of the Int
IO: Inferior olive
JC: Jaw-closing
JCMS: Jaw-closing muscle spindle
jcms-IntDL: Regions of IntDL receiving JCMS proprioceptive inputs
jcms-MedDL: Regions of MedDL receiving JCMS proprioceptive inputs
JO: Jaw-opening
KF: Kölliker-Fuse nucleus
Lat: Lateral cerebellar nucleus
LC: Locus coeruleus
Li: Linear nucleus of the medulla
LRF: Lateral reticular formation
LRt: Lateral reticular nucleus
LVe: Lateral vestibular nucleus
mcp: Middle cerebellar peduncle
Me5: Trigeminal mesencephalic nucleus
Med: Medial cerebellar nucleus
MedDL: Dorsolateral protuberance of the Med
ml: Medial lemniscus
Mo5: Trigeminal motor nucleus
MVe: Medial vestibular nucleus
Pa5: Paratrigeminal nucleus
PaSol: Parasolitary nucleus
PB: Phosphate buffer
Pb: Parabrachial nucleus
PM: Paramedian lobule
Pn: Pontine nucleus
Pr: Prepositus nucleus
Pr5: Trigeminal principal nucleus
py: Pyramidal tract
Rm5vm: Reticular region medial to the 5vm
RtTg: Reticulotegmental nucleus
scp: Superior cerebellar peduncle
Sim: Simple lobule
SO: Superior olive
Sol: Nucleus of the solitary tract
sp5: Trigeminal spinal tract
SpVe: Spinal vestibular nucleus
Su5: Supratrigeminal nucleus
SuVe: Superior vestibular nucleus
tth: Trigeminothalamic tract
Tz: Nucleus of the trapezoid body
un: Uncinate fasciculus of the cerebellum
unx: Decussation of the un
vsc: Ventral spinocerebellar tract
Y: Group y of the vestibular nucleus
